# scRNA-seq generates a molecular map of emerging cell subtypes after sciatic nerve injury in rats

**DOI:** 10.1038/s42003-022-03970-0

**Published:** 2022-10-19

**Authors:** Ditte Lovatt, Alex Tamburino, Alicja Krasowska-Zoladek, Raul Sanoja, Lixia Li, Vanessa Peterson, Xiaohai Wang, Jason Uslaner

**Affiliations:** 1 Department of Neuroscience, Merck & Co., Inc, West Point, PA USA; 2 Department of Data and Genome Sciences, Merck & Co., Inc, West Point, PA USA; 3 Department of Genome and Biomarker Science, Merck & Co., Inc, Boston, MA USA; 4 Present Address: Biomarkers & Imaging, Vertex Pharmaceuticals, Boston, USA

**Keywords:** Neuroscience, Transcriptomics

## Abstract

Patients with peripheral nerve injury, viral infection or metabolic disorder often suffer neuropathic pain due to inadequate pharmacological options for relief. Developing novel therapies has been challenged by incomplete mechanistic understanding of the cellular microenvironment in sensory nerve that trigger the emergence and persistence of pain. In this study, we report a high resolution transcriptomics map of the cellular heterogeneity of naïve and injured rat sensory nerve covering more than 110,000 individual cells. Annotation reveals distinguishing molecular features of multiple major cell types totaling 45 different subtypes in naïve nerve and an additional 23 subtypes emerging after injury. Ligand-receptor analysis revealed a myriad of potential targets for pharmacological intervention. This work forms a comprehensive resource and unprecedented window into the cellular milieu underlying neuropathic pain and demonstrates that nerve injury is a dynamic process orchestrated by multiple cell types in both the endoneurial and epineurial nerve compartments.

## Abstract (web summary)

Single-cell RNA-seq analysis of naive and injured sensory nerves in a rat model provide further insight into the molecular pathways underlying nerve injury, regeneration, and repair.

## Introduction

Neuropathic pain is caused by damage or disease to the peripheral somatosensory nervous system and is diagnosed when non-noxious temperature or tactile stimuli is experienced as painful. Frequent causes include metabolic, viral, or chemical disruption leading to debilitating conditions such as diabetic neuropathy, postherpetic neuralgia, and chemotherapy-induced neuropathic pain, respectively. Gabapentinoids, tricyclic antidepressants, opioids, and serotonin-norepinephrine reuptake inhibitors are among available treatments that demonstrate modest efficacy, however, they also produce a range of unwanted adverse effects limiting their use, including lethargy, nausea, and potential for addiction^1^. A common cellular target of analgesic therapies involve managing neuronal excitation directly without addressing dysfunctional signaling of nerve-resident non-neuronal cell types, which may be involved in precipitating neuropathic pain. Hence, studies exploring novel mechanisms of neuropathic pain and identifying strategies for interfering with non-neuronal peripheral signals could accelerate the development of long-needed therapies into the clinic^2^. Given the poor management of neuropathic pain by available treatments, this clearly defines an area of medical need.

The highly complex and heterogenous microenvironment of nerve tissue has historically challenged efforts pinpointing the molecular changes that accompany nerve injury with cellular resolution. In turn, this has compromised the ability to clearly identify cellular subtypes that reside within naive nerve and that emerge after injury, and has led to controversies regards the cellular origins of promising targets and how to experimentally validate them. For instance, controversy exists as to the cellular origin of the drug target AT2R^3,4^. Over the past decades only a limited number of novel drug targets have entered the clinic, only to fail in demonstrating robust efficacy. Therefore, in order to devise a clear and actionable strategy for the discovery of novel analgesics, we must obtain a better understanding for the molecular changes in nerve after insult and the cellular underpinnings that may develop into persistent neuropathic pain.

Recently, the development of single-cell RNA-sequencing (scRNA-seq) has allowed for detailed discovery of cell subtypes in tissues and have enabled a substantial expansion of cell type classification in many tissues, including the CNS^5^. Application of scRNA-seq to nerve tissue specimens^6–9^ have recently provided novel insight into the cellular composition of nerve tissues. However, the limited sizes of these studies prevented in depth identification of any scarce cell subtypes that reside in naive nerve or arise after insult.

Here, in our study, we analyzed high-quality transcriptomes in more than 112,000 individual cells from sciatic nerve tissue, which enabled deciphering the cellular composition of naive nerve among five major cell groups including Schwann cells, fibroblasts, lymphoid cells, myeloid cells, and vascular cells. Further, we also investigated the molecular consequences after injury using the chronic constriction injury (CCI) model^10–12^ in which rats develop chronic neuropathic pain within days of injury. Our study reports on the identification of 45 different cell subtypes in naive tissue, and 23 transient or persistent cell subtypes that emerge after injury, and reveals the ligand–receptor interactions among them. This study provides a foundational resource for the exploration of cellular interactions that accompany nerve injury, and may inspire future studies exploring novel drug targets as well as the causality that underlie the emergence and persistence of nociception in painful conditions.

## Results

**Fig. 1:**
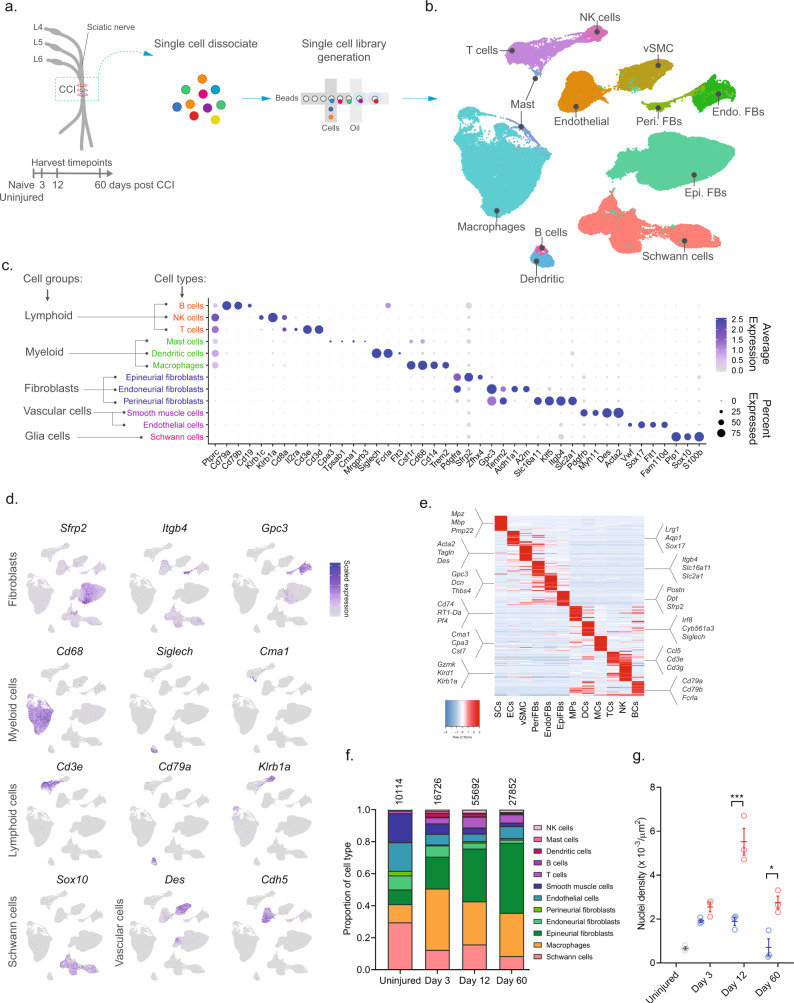
Classification of major cell types within rat sciatic nerve. **a** Experimental outline of dissociation of sciatic nerve from naive animals and from CCI injured animals at 3, 12, and 60 days post-injury. **b** UMAP plot of clustered cells from all timepoints (*n* = 112,521 cells, *n* = 33 10X libraries, *n* = 18 animals, *n* = 4 timepoints). **c** Dot plot of scaled expression of genes differentiating major cell types and classification of their group. **d** Feature plot of cell type markers. Cells plotted in random order. **e** Heat map displaying scaled expression of top 20 differentially expressed genes among major cell types using cluster average expression values. **f** Proportion of cell types in scOmics data at each timepoint. **g** Nuclei density in nerve tissue at each timepoint quantified by histology (blue = contralateral, red = ipsilateral, gray = uninjured, individual values and mean ± SD, *n* = 5 technical replicates per animal, *n* = 3 animals, two-way ANOVA with Sidak’s multiple comparisons test, ***p < 0.0001, *p < 0.0035).

To investigate the cellular subtypes in naive nerve and those that emerge after nerve injury, we used scRNA-seq of enzymatically dissociated naive and injured rat sciatic nerve at three discrete timepoints post chronic constriction injury (CCI; 3, 12, and 60 days post CCI) (Fig. 1a). We chose these timepoints based on the development of mechanical allodynia and thermal hyperalgesia in the CCI model present at day 3, 12, and 60 post-injury^10–12^. At day 3 and 12, early and late phases of acute inflammation, respectively, is also relevant for the development of edema, cell infiltration, and proliferation. At day 60 post-injury, acute inflammation have subsided, some animals may experience a partial recovery of nociceptive hypersensitivity, but scarring of nerve persists. To ensure only tissue-adherent cells were profiled, but not circulating cells, we transcardially perfused the animals before tissue extraction, which resulted in a near elimination of red blood cells indicative of removal of circulating blood cells (Supplementary Fig. 1). From each timepoint, we sampled between 10,351 and 62,101 cells, and between 3 and 7 biological replicates (Supplementary Table 1) totaling 112,521 cluster-assigned cells for the entire dataset. We achieved high-quality data enabling us to identify ~2600 median genes per cluster across the entire dataset (Supplementary Fig. 1). Given the high number of cells and multiple timepoints assayed, our annotation strategy consisted of first using unsupervised clustering of all cells from all timepoints to classify every cell as a member of a major cell group (Fig. 1b), and then subsequently re-cluster individual major cell groups to explore biology within them.

Unsupervised clustering identified 32 clusters (Supplementary Figs. 2 and 3) that based on known markers and differentially expressed genes could be classified into five major cell groups described as lymphoid cells (B cells, NK cells, and T cells), myeloid cells (dendritic cells, mast cells, and monocytes), fibroblasts (epineurial, perineurial, and endoneurial fibroblasts), vascular cells (smooth muscle cells and endothelial cells), and Schwann cells (Fig. 1c–e). Using this approach, we classified >99% of the cells. Each cell type comprised cells from all timepoints, except for a very small group of cells which we termed mast cells based on markers expressed in the uninjured state (Supplementary Fig. 4). The resulting identification of cell types was consistent with previously published descriptions of the cellular anatomy of sciatic nerve, suggesting our protocols recovered cells irrespective of type. We did not recover neurons, for which the cell body reside in the dorsal root ganglion and were not within scope of the current effort and have been published elsewhere^13^.

In the naive nerve, we identified 36.1% vascular cells, 29.6% Schwann cells, 20.6% fibroblasts, 11.5% myeloid cells, and 2.2% lymphoid cells. While every major cell type was represented at each timepoint, there were significant changes in the proportion among them after injury (Fig. 1f). Importantly, using histology we determined that a significant increase in cell density was elicited by injury at days 12 and 60 (Fig. 1g), which may be explained by infiltration and proliferation of cells and should factor into the interpretation of relative proportion of type in Fig. 1f.

### Schwann cells respond to injury by proliferation and differentiation into a transient repair-like phenotype

**Fig. 2:**
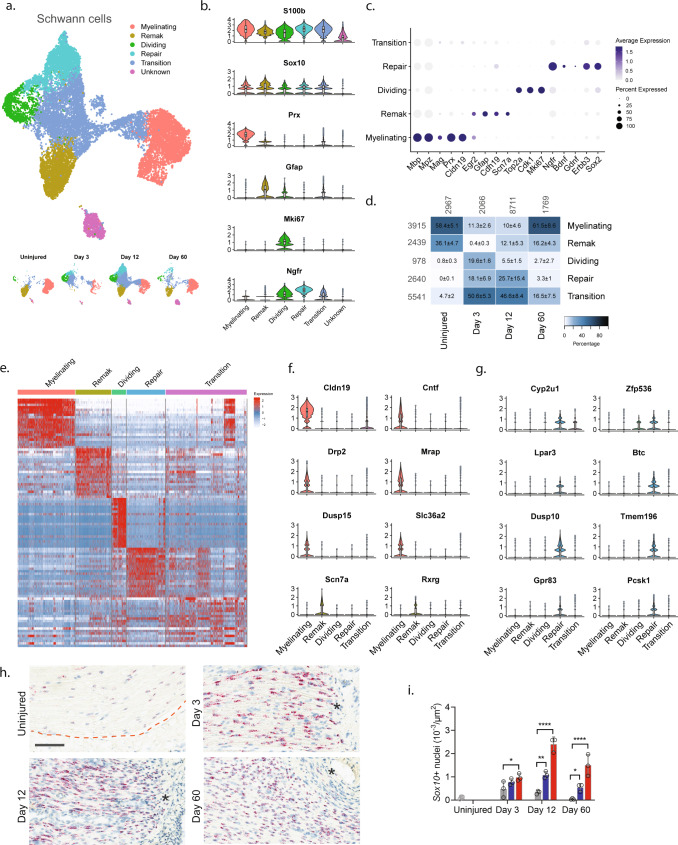
Identification of molecular subtypes of Schwann cells in naive and injured nerve. **a** UMAP plot of Schwann cells from all timepoints (*n* = 16,259 cells). Top: All timepoints. Bottom: Individual timepoints. **b** Violin plot with boxplot overlay and outliers (dots) of markers identifying Schwann cell subtypes across all timepoints. **c** Dot plot of scaled expression of markers identifying subtypes. **d** Heat map of the distribution (percentage) of each subtype normalized to each timepoint. Mean ± SD superimposed on each cell. Left row labels and top column labels indicate cell number in cluster and timepoint, respectively. **e** Heat map displaying scaled expression of top 20 differentially expressed genes among Schwann cell subtypes across all timepoints. Expression is scaled. **f** Violin plot with boxplot overlay and outliers (dots) of specific markers identifying myelinating and Remak Schwann cell subtypes among Schwann cells. **g** Violin plot with boxplot overlay and outliers (dots) of specific markers identifying Repair Schwann cell subtypes among Schwann cells. **h** ISH of *Sox10* RNA on uninjured and injured (3, 12, and 60 days post CCI) nerve. Dashed line indicates the perineurial barrier with the endoneurium above. Injured timepoints show only endoneurium and asterisks indicate ligature-induced necrosis. Scale bar, 100 µm. **i** Bar chart with dots of *Sox10* + nuclei density in uninjured (dark gray), contralateral (light gray), ipsilateral whole area (blue), and ipsilateral proximal to necrotic area (red) (*n* = 3 animals, mean ± SD, two-way ANOVA with Dunnett’s multiple comparison test, *p < 0.05, **p < 0.01, ****p < 0.0001).

To explore biology within cellular subtypes in each major cell group including cell proliferation and infiltrating types before and after injury, we next subclustered individual cell groups across all timepoints. We first subclustered the Schwann cell group (16,259 cells) and identified 15 clusters all of which expressed pan-Schwann cell markers (*S100b* and *Sox10*), except for cluster 9 (746 cells) which comprised putative doublets (Fig. 2a and Supplementary Fig. 5). Based on known markers, we were able to further classify each cluster into one of the following subtypes: myelinating Schwann cells (*Prx*^14^), non-myelinating Remak Schwann cells (*Gfap*^15^), dividing Schwann cells (*Mki67*^16^), repair cells (*Ngfr*, also known as p75NTR^17^), as well as a cluster of cells we termed transition cells (Fig. 2b, c and Supplementary Fig. 5). All the Schwann cell subtypes expressed specific gene signatures further supporting they are subtypes, except for the transition type, which likely consisted of multiple transitional phenotypes with a temporal signature (Fig. 2d, e).

In naive nerve, 94% of Schwann cells classified as either myelinating (58.4%) or non-myelinating Remak Schwann cells (36.1%) (Fig. 2d). We furthered identified 0.8% dividing Schwann cells and 4.7% transitional cells suggesting that ~1% of Schwann cells undergo homeostatic renewal in naive nerve. We also identified several markers that were selective to naive Remak and myelinating Schwann cells, respectively, compared to all Schwann cell subtypes and all major cell types across all timepoints. In myelinating Schwann cells, we identified 14 markers which included *Prx* and several other markers harboring a more selective profile including *Cldn19*, *Cntf*, *Drp2*, *Mrap*, *Dusp15*, and *Slc36a2* (Fig. 2f and Supplementary Fig. 6). Surprisingly, while *Gfap* could be identified as a Remak subtype marker, we failed to identify any markers with properties better than *Gfap* in terms of higher selective expression compared to other Schwann subtypes and all major cell groups. However, markers that were similar to *Gfap* included *Rxrg*, *Grin2b*, *Fxyd7*, *Ninj2*, and *Scn7a*^18^ (Fig. 2f and Supplementary Fig. 6).

Interestingly, Schwann cell clusters displayed strong temporal patterning, suggesting that injury triggers molecular changes in Schwann cells. In particular, injury induced the emergence of a transient repair type and a steep increase among dividing Schwann cells (Fig. 2a, d). At day 3 after injury, there was a significant change in the proportion of Schwann cell subtypes including an increase in dividing cells from ~1% to ~20%. This was consistent with histological analysis demonstrating an increase in the density of *Sox10* + nuclei within the endoneurium after injury (Fig. 2h, i). The surge of dividing cells coincided with emergence of a neighboring cluster to the dividing one. This injury-specific subtype comprised ~18% of all Schwann cells at day 3 and expressed *Ngfr* consistent with previously described repair cells^19,20^. These injury-specific repair cells could be distinguished from others by many differentially expressed genes (Fig. 2e, g and Supplementary Fig. 7). While repair cells were abundant after injury, only one cell among all naive Schwann cells classified as a repair cell, emphasizing that the repair cluster was injury specific. However, the repair subtype was only transient, emerging at 3 days (~18%), persisting at 12 days (~26%), and only sparsely present at 60 days (~3%). Interestingly, dividing cells followed a similar pattern (0%, ~20%, ~6%, and ~2% at naive, 3, 12, and 60 days post injury, respectively). At day 60 post-injury, the proportion of Remak and myelinating Schwann cells had been partially replenished, suggesting that injury triggers cell division and a transient repair cell type in Schwann cells.

We next looked for differentially expressed markers that would selectively label repair cells in injured nerve. Among the 15 most selective genes, we identified several with a known role in neuropathic pain (*Cyp2u1* and *Lpar3*^21^), or in injured and differentiating Schwann cells (*Zfp536*^22^*, Btc,* and *Pcsk1*^23^), while other selective genes had a more subtle profile associated with nociception (*Gpr83*^24,25^, *Dusp10*, and *Tmem196*) (Fig. 2g and Supplementary Fig. 7). We also identified a significant increase in genes previously described^17,26^ in repair cells including *Ngfr*, *Bdnf*, *Erbb3*, and *Sox2* (*p* < 6.4 × 10^−214^, Fig. 2e) further establishing that these cells are repair cells. Altogether, our data show that in naive injured nerve most Schwann cells comprise Remak or myelinating cells which undergo a low level of renewal during homeostasis. After injury, however, a dramatic increase is evident in cell renewal, which is followed by the emergence of a transient repair cell type at which the nerve undergo elimination of necrotic cells and remodeling.

### Anatomically restricted fibroblasts display distinct phenotypical changes after injury

**Fig. 3:**
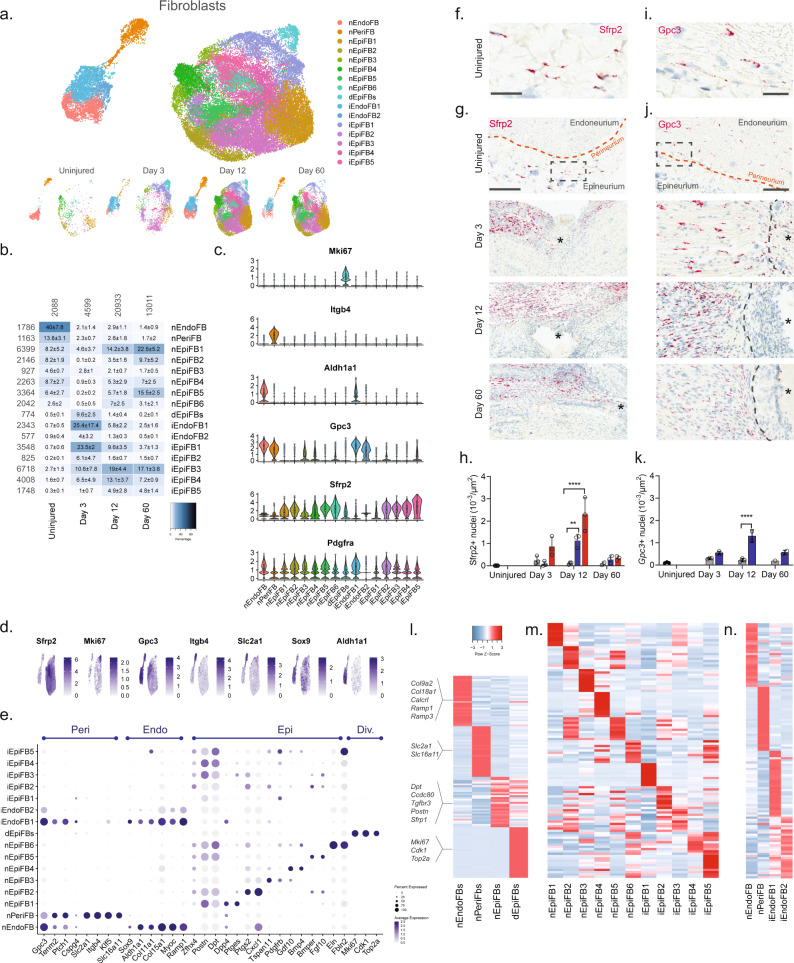
Identification of molecular subtypes of fibroblast cells in naive and injured nerve. **a** Dimensional reduction plot (UMAP) of subclustered fibroblasts from all timepoints (*n* = 40,361 cells). Top: All timepoints. Bottom: Individual timepoints. **b** Heat map of the distribution of fibroblast subtypes at each timepoint (percentage, mean ± SD). Left row labels and top column labels indicate cell number in cluster and timepoint, respectively. **c** Violin plot with boxplot overlay and outliers (dots) of fibroblast subtype markers. **d** Feature plot of key markers differentiating epi-, peri- and endoneurial fibroblasts. **e** Dot plot of scaled expression of fibroblast subtype markers. **f**, **g** ISH of *Sfrp2* (Scale bar: **f**, 75 µm; **g**, 300 µm) and **i**, **j** *Gpc3* (Scale bar: **i**, 25 µm; **j**, 100 µm) in uninjured and injured (3, 12, and 60 days post-CCI) sciatic nerve. Asterisk: Necrotic area. The dashed boxes in (**g**) and (**j**) display the image in (**f**) and (**i**), respectively. **h**, **k** Bar chart with individual values displaying ISH data in (**g**) and (**j**) in uninjured (dark gray), contralateral nerve (light gray), ipsilateral - whole area (blue), and ipsilateral - epineurial area (red) for each timepoint (mean ± SD, *n* = 2–3 animals, two-way ANOVA with Sidak’s multiple comparison test, **p < 0.01, ****p < 0.0001). **l**, **n** Heatmaps of top differentially expressed genes in: **l** the four major fibroblast types, **m** naive and injured epineurial subtypes, and **n** naive and injured peri- and endoneurial subtypes. Normalized Z-score.

We next subclustered all fibroblasts (40,361 cells) and identified 23 clusters all of which expressed *Pdgfra* (Fig. 3a, b and Supplementary Fig. 8), except for one cluster which we later identify as injured endoneurial fibroblasts (iEndoFBs type 2577 cells).

We first classified each of the 23 clusters into major cell types based on markers associated with the three subanatomical compartments within nerve: epineurial (*Sfrp2*), perineurial (*Gpc3*, *Itgb4*, and *Slc2a1*), and endoneurial (*Gpc3, Sox9*, and *Aldh1a1*) fibroblasts in agreement with previous studies^6,27^ (Fig. 3c–e and Supplementary Fig. 8). Further, a subset of the epineurial fibroblasts were labeled as dividing (*Sfrp2* and *Mki67*^16^). We confirmed the spatial patterning of these three major fibroblast types in nerve by histology which demonstrated that *Sfrp2* exclusively labeled cells in the epineurium, and *Gpc3* exclusively labeled cells in the endoneurium and perineurium, respectively (Fig. 3f–k). These three anatomical types were clearly distinguished by dimensional reduction, regardless of injury state, and displayed differential gene expression when compared to one another (Fig. 3a, l).

In uninjured naive nerve, ~93% of fibroblasts classified as either perineurial (~41%), endoneurial (~13%), or epineurial (~36%) fibroblasts. The remaining cells included dividing and transitional epineurial types. Perineurial fibroblasts establish a physical barrier shielding the endoneurial compartment from the environment outside the nerve. Supporting a role in this function, we identified that perineurial cells expressed several metabolic transporters including glucose (*Slc2a1*) and monocarboxylate (*Slc16a11*) transporters (Fig. 3e, l). Endoneurial fibroblasts on the other hand expressed collagens supporting extracellular matrix maintenance (*Col9a2*, *Col11a1*, *Col15a1*, and *Col18a1*), receptors for CGRP signaling (*Calcrl*, *Ramp1*, *Ramp3*), and *Sox9* suggesting a role in stemness^28^.

In contrast, to the limited types of peri- and endoneurial fibroblasts, epineurial fibroblasts displayed much broader diversity. To this end, we identified six subtypes of epineurial fibroblasts in naive nerve based on dimensional reduction and differential gene expression (Fig. 3a, m).

Interestingly, nEpiFB (naive epineurial fibroblast) type 1 was enriched for genes (*Dpp4*, *Ptges* (PGE2), and *Car8*^29–31^) with a role in inflammation, while nEpiFB type 2 was enriched for triggers of pain and neutrophil recruitment (*Ptgs2* (Cox-2), *Cxcl1*^32^, and *Irf1*^33^) (Fig. 3m). Subtype nEpiFB type 3 expressed many genes involved in development (*Tspan11, Spry1*^34^*, Cldn1*^35^), while type 4 expressed genes with a role in differentiation of progenitors (*Gdf10*^36^, *Bmp4*^37^). Type 5 expressed genes with known function in regulating mesenchymal mobilization and angiogenesis (*Bmper*^38^ and *Fgf10*^39,40^), as well as Neurotrophin signaling in regeneration (*Ntrk2*^41^, *Ntf3*^42^), positioning these cells with a role in axonal regeneration and nerve repair. The last type, type 6, expressed genes involved in extracellular matrix formation including fibulin, elastin, collagen, and osteoglycin (*Fbln2*^43^, *Eln*^44^, *Col8a1*, *Ogn*^8^). Injury triggered a sharp change in the proportion of cells across the different subtypes compared to naive which in part was due to cell proliferation or infiltration (Fig. 3b). At day 3, the relative proportion of dividing cells among all fibroblast types had increased from 0.5% to ~10%. This coincided and preceded a significant increase in the density of *Sfrp2* + cells of ~3, ~21, and ~4 fold at day 3, 12, and 60 post-CCI, respectively, when examined by histology (Fig. 3h). Similarly, the density of *Gpc3* + cells increased ~2, ~6, and ~3 fold at day 3, 12, and 60 post-CCI, respectively (Fig. 3k). Intriguingly, the proportion of endoneurial fibroblasts decreased from ~40% to only ~2% at day 3. This coincided with the emergence of two previously sparsely populated iEndo types. Type 1, which increased from ~1% in naive to 18%, 5%, and 2% at day 3, 12, and 60 post-CCI, respectively, expressed genes involved in differentiation and wound repair (*Sox8, Bmp7, Igsf3*^45^*, Fgf2r*^39^*, Ptprn*) (Fig. 3n). iEndo type 2, which changed from ~1% to 5%, 1%, and 0.5% at day 3, 12, and 60 post-CCI, respectively, display differential expression of tens of ribosomal proteins.

Distinct epineurial types also emerged after injury with some differences in the temporal signature when they emerged (Fig. 3b). iEpiFB type 1 cells emerged robustly from ~0.5% in naive nerve to ~24%, ~9%, and ~4% at day 3, 12, and 60 post-CCI, respectively. This cluster was the only neighbor to dEpiFBs in the dimensional reduction plot suggesting that this transient type were newly formed cells. iEpiFB type 1 cells expressed genes involved in fibrosis (*Acta2, Des, Tnc*^46^), neuromodulator signaling (*Npy1r*^47^*, Tac3*^48^), and neutrophil chemokine signaling (*Cxcl6*) (Fig. 3m). iEpiFBs type 2 and type 3 had some overlap of genes involved in B cell, neutrophil, and monocyte chemotaxis as well as hematopoietic stem cells migration (*Pdgfra*, *Tnfsf13b*^49^, *Cxcl12*^50^, *Ccl7*^51^). iEpiFBs type 4 and type 5 appeared to share some expression with nEpiFBs type 6 in addition to also expressing genes involved in remodeling (*Mmp11, Mmp14, Col8a1, Col11a1, Cilp*^52^*, Cilp2*).

By subclustering ~40,000 nerve fibroblasts, we have identified nine subtypes of naive fibroblasts among three subanatomical regions within nerve. After injury we further identified the emergence of seven different types of fibroblasts based on genes with roles supporting immune infiltration, activation, and modulation as well as tissue remodeling. Altogether, our findings highlight the complexity of fibroblast heterogeneity, and shed light into their contribution to homeostasis and repair after nerve injury.

### Injury triggers myeloid cell differentiation and infiltration, and the generation of a myeloid scar

**Fig. 4:**
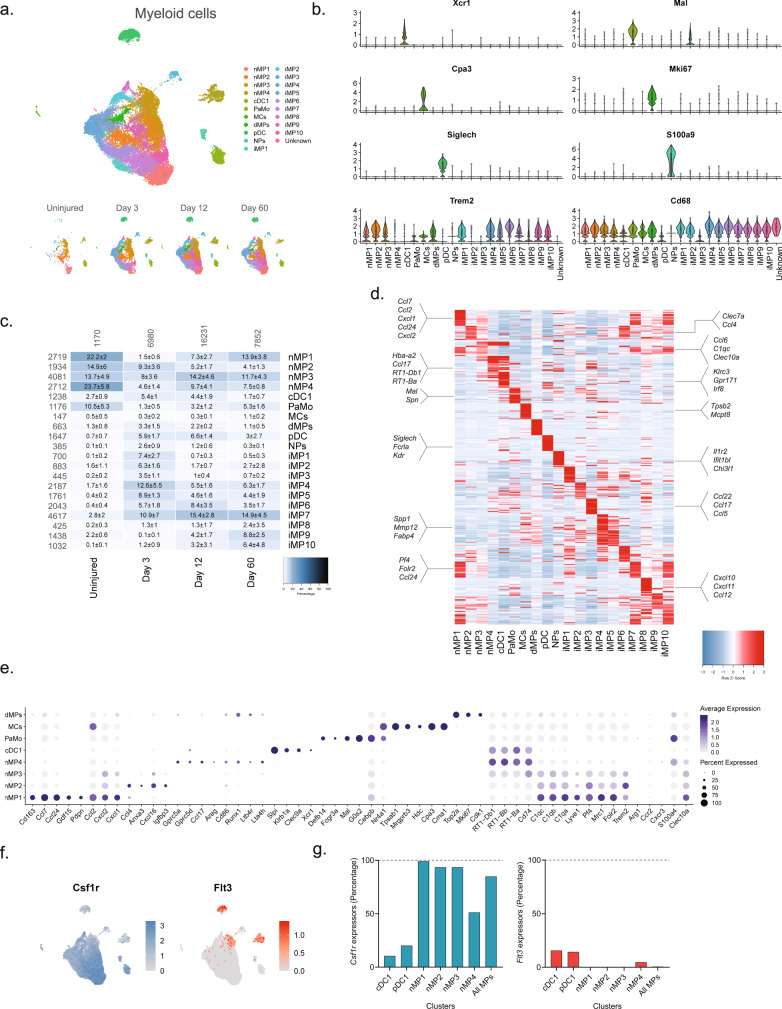
Identification of molecular subtypes of myeloid cells in naive and injured nerve. **a** Dimensional reduction plot (UMAP) of subclustered myeloid cells (*n* = 32,265 cells). Top: All timepoints. Bottom: Individual timepoints. **b** Violin plot with boxplot overlay and outliers (dots) of cell type markers. **c** Heat map of the distribution of each myeloid subtype at each timepoint (percentage, mean ± SD). Left row labels and top column labels indicate cell number in cluster and timepoint, respectively. **d** Heat map displaying scaled expression of the top 40 differentially expressed genes across cell types from all timepoints. **e** Dot plot of scaled expression of genes differentiating clusters in naive and injured dominant subtypes. **f** Feature plot of Csf1rand Flt3 expression distribution across cells from all timepoints, respectively. **g** Quantification of the percentage of cells expressing (*Csf1r*, left) and (*Flt3*, right) in relevant clusters.

We next subclustered all myeloid cells (32,265 cells) which identified 24 clusters all of which expressed the pan-myeloid marker *Cd68*, except for cluster 19 (386 cells) (Fig. 4a, b and Supplementary Fig. 9). Based on known markers, we were able to classify clusters in naive nerve into the following subtypes: conventional dendritic cells type 1 (cDC1s, *Xcr1*^53^, and *Clec9a*^54,55^), mast cells (*Cpa3*^56^ and *Cma1*^57^), patrolling monocytes (*Nr4a1*^58,59^), dividing macrophages (*Mki67*^16^), and macrophages (nMPs1-4, *Trem2*) (Fig. 4b–e). Macrophages could be subdivided further into either complement expressing (*Cd163*^60^*, C1q*^61^) or MHC class II expressing (*RT1*^62^ genes). The complement expressing cells could be divided into two clusters based on differential expression of *Cd163*, *Ccl7*, *Ccl24*, *Ccl2*, *Cxcl1*, and *Cxcl2* (nMP1, naive macrophages type 1), or *Ccl4*, *Anxa3*, *Ifngr1*, *Cxcl16*, and *Igfbp3* (nMP2) suggesting their involvement in neutrophil and monocyte recruitment. A third cluster of naive macrophages (nMP3) expressed both complement and MHC II molecules, and could not be distinguished based on additional selective molecules, suggesting their phenotype was less specialized. A fourth cluster (nMP4) could be identified by expression of MHC II genes (*RT1-Db1, RT1-Bb, RT1-Ba*^63^), consistent with previous studies^7^. nMP4 also expressed several pro-inflammatory mediators (*Ccl17*) and enzymes and receptors for leukotriene B4 signaling (*Ltb4r* and *Lta4h*) suggesting their involvement in neutrophil and leukocyte recruitment.

Interestingly, the naive macrophage clusters displayed a distinct pattern of Clec10a and S100a4, markers which in another study labeled epineurial macrophages and recently recruited monocyte-derived macrophages, respectively^9^, suggesting that nMP1-3 are epineurial and nMP4 are recruited. Intriguingly, the nMP4 cluster also displayed expression of two different myeloid lineage markers, *Flt3* and *Csfr1*, indicating that unlike other nMP types (or injury types) which did not express *Flt3*, the nMP4 phenotype is populated by macrophages deriving from both embryonic precursors and bone marrow lineages (Fig. 4f). In dendritic cells (cDC1 and pDC1), which derive from the hematopoietic lineage, Flt3 could be detected in about 15% of cells suggesting this gene could be underestimating the percentage of hematopoietic derived cells (Fig. 4g). While this result suggests that most nerve macrophages (~85%) derive from early development in line with previous studies^9^, it also shows that both lineages can differentiate into one phenotype as determined by dimensionality reduction. Thus, here we describe four transcriptionally distinct subdivisions of tissue-resident naive macrophages, adding two previously uncharacterized types, and further identify evidence suggesting that the MHC II macrophage phenotype can be populated by different lineages.

**Fig. 5:**
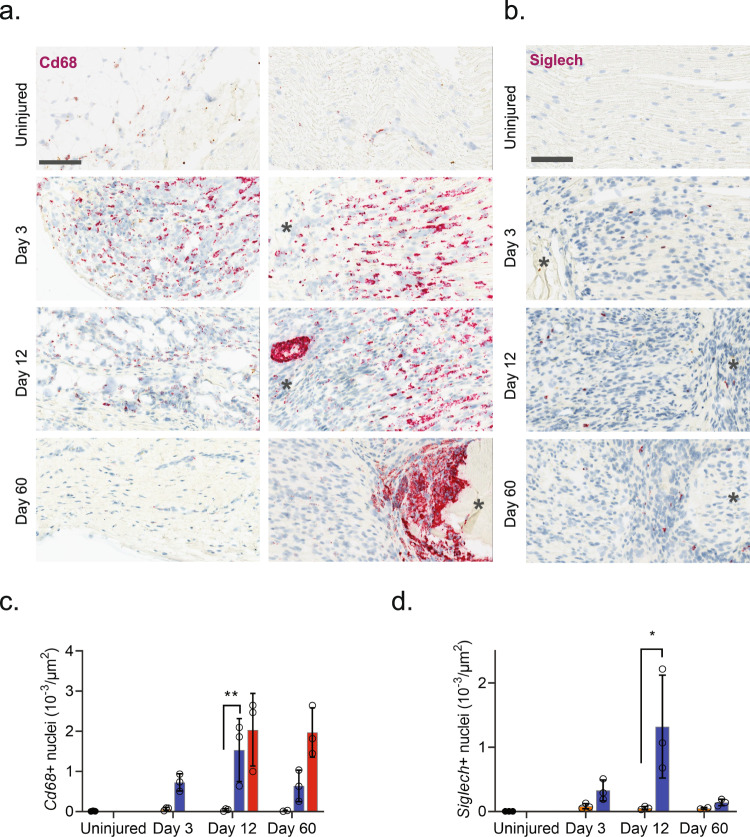
Myeloid cell types form a enveloping scar around necrotic areas. **a** ISH of *Cd68* in uninjured and injured (3, 12, and 60 days post-CCI) sciatic nerve. Column: left, epineurium; right, endoneurium. Asterisk: Necrotic area. Scale bar, 100 µm. **b** ISH of *Siglech* in endoneurium of uninjured and injured (3, 12, and 60 days post-CCI) sciatic nerve. Asterisk: Necrotic area. Note that at day 3 nerve does not exhibit necrosis. Scale bars: 100 µm. **c**, **d** Bar chart with individual values of ISH data in (**a**) and (**b**) in uninjured (dark gray), contralateral nerve (light gray), ipsilateral—whole area (blue), and ipsilateral—necrotic area (red) for each timepoint (mean ± SD, *n* = 3 animals, two-way ANOVA with Sidak’s multiple test comparison: *p < 0.05, **p < 0.01).

Injury triggered the emergence of several distinct myeloid subtypes driven by phenotypical changes or division of resident cells, and infiltration of additional cell types (Fig. 4b). Histological analysis revealed a dramatic increase in the density of *Cd68* + cells after injury which subsided toward naive levels at 60 days, except for highly necrotic areas which remained encapsulated by Cd68+ cells (Fig. 5a, b). At three days after injury several clusters emerged including plasmacytoid dendritic cells (pDCs, *Siglech*^64^) and neutrophils (*S100a8*^65^*, S100a9*^65,66^) as expected at this timepoint. To verify that this was not just a confound of dissociation technique, we inspected the density of *Siglech* + cells in nerve. Our analysis revealed the emergence of pDCs cells which peaked at day 12 post injury, consistent with observations in the single-cell data (Contra- vs. ipsilateral at days 3, 12 and 60, respectively: 0.08 ± 0.04 vs. 0.33 ± 0.16, 0.05 ± 0.03 vs. 1.32 ± 0.80, and 0.005 ± 0.001 vs. 0.14 ± 0.05 cells (10^−3^/µm^2^), n = 2–3 animals) (Fig. 5c, d). The emergence of neutrophils and pDC1s was accompanied by numerous injury-specific clusters of macrophages some of which appeared transiently while other were more persistent over the time course investigated (Fig. 4c). These clusters were distinguishable based on markers and differentially expressed genes (Fig. 4d).

The emergence of injury-specific myeloid subtypes is consistent with the temporal mechanics of inflammatory initiation and resolution in other tissues^67^. Consistent with these studies we identify the emergence of several transient iMPs (injury-enriched macrophages) and neutrophils at the early timepoint. Injury caused the emergence of neutrophils which could be detected at 3 days after injury. These cells expressed many molecules consistent with a pro-inflammatory profile, including *Il1b*, *Ccl3*, and *Cxcr2* expression as well as enzymes and receptors for leukotriene B4 signaling (*Ltb4r* and *Lta4h*) consistent with autocrine signaling^32,67–70^ (Fig. 4d).

However, surprisingly we did not identify a clearly defined cluster with high expression of *Ccr2* (Fig. 4e), a factor necessary for monocyte extravasation and inflammatory resolution in other tissues, aside from very scarce expression in iMP2s^71^. Previous studies in rodent nerve have shown that Wallerian degeneration can progress independently of *Ccr2* suggesting that nerve might be a specialized tissue where *Ccr2* monocyte extravasation does not play a crucial role in inflammation, that Ccr2 is expressed in neurons not captured by our study, or we missed the temporal window for identifying these cells in our studies^72^.

The appearance of later arriving iMP types is also consistent with the phenotypical adoption of tissue repair programs in resident MPs. In other tissues these include an early appearance of a pro-inflammatory iMP which help control neutrophil life span followed by a phagocytotic iMP type which help remove debris and apoptotic neutrophils. Histological inspection of *Cd68* + cells revealed a dramatic increase in myeloid cells after injury and a distinct and temporally dependent distribution of Cd68+ cells within the tissue. This was evident by widespread homogenous distribution of Cd68+ cells within nerve at 3 days after injury. However, at 60 days after injury CD68 + cells displayed a distinct spatial pattern restricted to enveloping necrotic areas (Fig. 5a).

### Vascular cells display change after injury with minimal injury enriched subtypes emerging

**Fig. 6:**
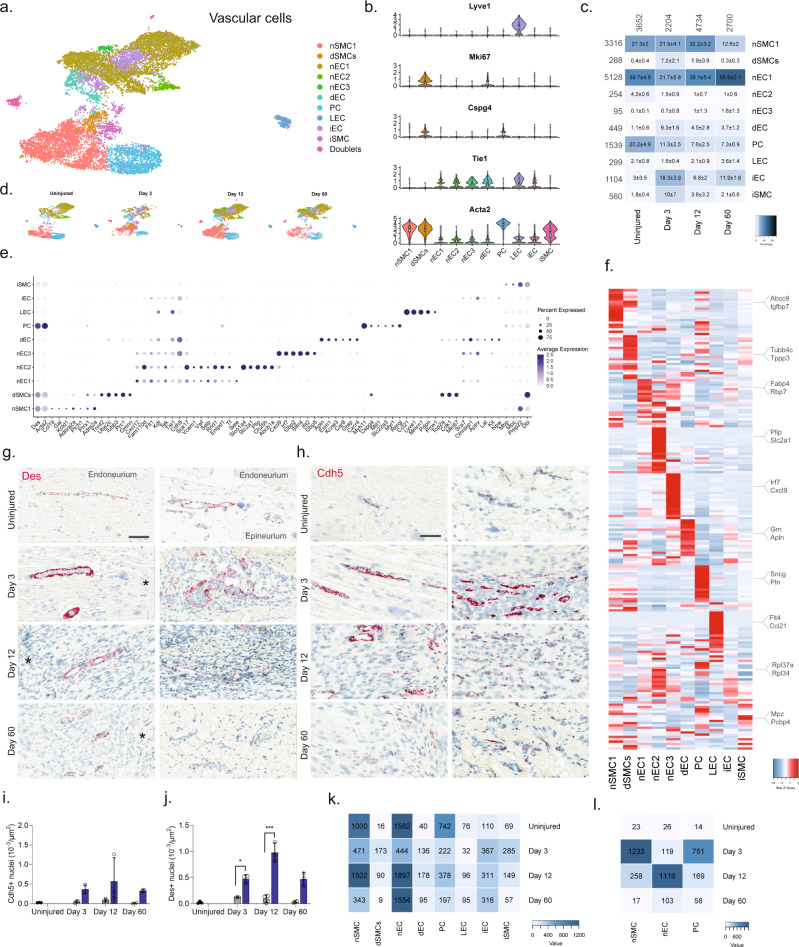
Identification of molecular subtypes of vascular cell types in naive and injured nerve. **a** Dimensional reduction plot (UMAP) of subclustered vascular cells from all timepoints (*n* = 13,290 cells). Top: All timepoints. Bottom: Individual timepoints. **b** Violin plot with boxplot overlay and outliers (dots) of cell type markers. **c** Heat map of the distribution of each vascular subtype at each timepoint (percentage, mean ± SD). **d** Feature plots of vascular subtype markers. **e** Dot plot of scaled expression of genes differentiating clusters in among naive and injured subtypes. **f** Heat map displaying scaled expression of the top 20 differentially expressed genes across cell types from all timepoints displayed from cluster averages. **g** ISH of *Des* in endoneurium (left column) and epineurium (right) of uninjured and injured (3, 12, and 60 days post-CCI) sciatic nerve. Asterisk: Necrotic area. Scale bars: 100 µm. **h** ISH of *Cdh5* in endoneurium (left column) and epineurium (right) of uninjured and injured (3, 12, and 60 days post-CCI) sciatic nerve. Asterisk: Necrotic area. Scale bars: 100 µm. **i**, **j** Quantification of ISH data in (**g**) and (**h**), respectively, displayed as bar charts with individual values: uninjured (dark gray), contralateral nerve (light gray), and ipsilateral—whole area (blue) for each timepoint (mean ± SD, *n* = 2–3 animals, two-way ANOVA with Sidak’s multiple comparison, *p < 0.05, ***p < 0.001). **k** Heat map of cell count in each cluster divided by timepoint. **l** Heat map of absolute count of DEGs for each cell subtype when compared among timepoints. Only cell subtypes with more than 150 cells in each group were included in the analysis.

We next subclustered vascular cells (13,290 cells) into 17 clusters, which all strongly expressed markers^73^ for vascular smooth muscle cells (vSMC: *Acta2*, but no *Cspg4*), pericytes (*Acta2* and *Cspg4*), or endothelial cells (EC: *Tie1*), except for cluster 14 which expressed *Acta2* and *Tie1* weakly, but in addition also expressed markers for epineurial fibroblasts (*Sfrp2*, *Pdgfra*, *Zfhx4*), prompting us to annotate this cluster as doublets (Fig. 6a–d and Supplementary Fig. 10). EC marker analysis further identified clear clusters for dividing endothelial cells (*Tie1* and *Mki67*^16^) as well as lymphatic endothelial cells (LEC: *Pdpn*^74^, *Lyve*, *Ccl21*^75^, *Prox1*^76^). The expression of *Ccl21* in LECs is consistent with their role in shuttling neutrophils, dendritic, and T cells to the tissues, and play a role in inflammation. The remaining ECs could be divided into three clusters distinguished by markers (Fig. 6e, f) with nEC1 being the most abundant EC cluster. While we did not investigate the subanatomical relationship of these three clusters, we did, however, observe interesting markers suggesting functional roles that could impact drug uptake: nEC2 expressed Abcb1a, an ATP-dependent efflux pump with broad substrate specificity also known as Pgp suggesting that capillary vessels in either the epi- or endoneurial compartment may decrease drug penetration similar to the blood–brain barrier in the CNS. nEC3 expressed several markers (*Gpb2*, *Gbp5*, *Ifit2*) suggesting a role for this subtype in response to interferon-gamma stimulation^77^. In contrast to other major cell groups, injury did not seem to trigger the emergence of distinct cell states as evaluated by dimensional reduction. Instead, only one type of ECs (iECs) and one type of vSMC (iSMC) emerged after injury (Fig. 6c). The majority of top DEGs in iECs were mostly ribosomal protein genes (e.g., *Rpl34* and *Rps15a*) and iSMC expressed a less specific profile (Fig. 6f). This prompted us to investigate whether changes occurred within clusters and across timepoints. For this analysis, we only included nECs (nEC1-3), nSMC, and PC all of which had at least 150 cells represented at each timepoint (Fig. 6k), and we identified that these cell types demonstrated increased number of DEGs at Day 3 or Day 12 compared to the uninjured (Fig. 6l). Injury also triggered a 5–6 fold increase in the proportion of both dividing ECs (dECs: *Tie1* and *Mki67*) and SMC (dSMC: *Acta2*, *Cspg4*, *Mki67*) (Fig. 6c), which is supported by an increase in the density of both ECs and SMCs when examined by histology (Fig. 6g, j). In summary, vascular cells displayed the least diversity after injury responding with robust proliferation rather than the emergence of injury-specific subtypes.

### Unlike T cells, NK cells infiltrate primarily the epineurial nerve compartment

**Fig. 7:**
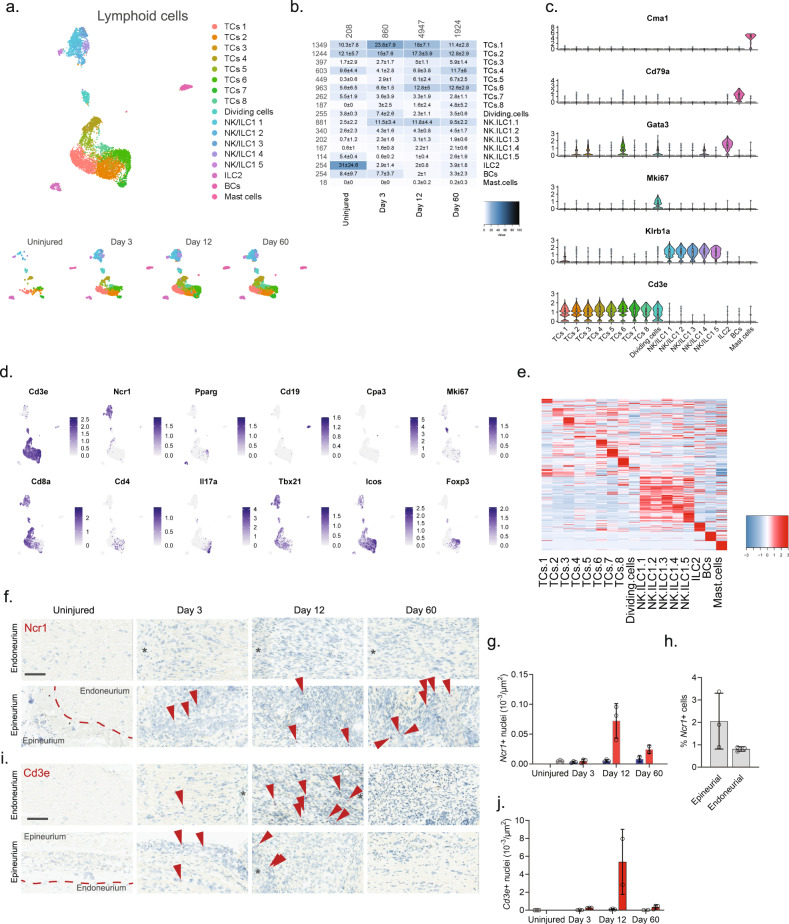
Identification of molecular subtypes of lymphoid cells in naive and injured nerve. **a** Dimensional reduction plot (UMAP) of subclustered lymphoid cells from all timepoints (*n* = 7957 cells). Top: All timepoints. Bottom: Individual timepoints. **b** Heat map of the distribution of each lymphoid subtype at each timepoint (percentage, mean ± SD). Left row labels and top column labels indicate cell number in cluster and timepoint, respectively. **c** Violin plot with boxplot overlay and outliers (dots) of cell type markers. **d** Feature plots of lymphoid subtype markers. **e** Heat map displaying scaled expression of the top 20 differentially expressed genes across cell types from all timepoints displayed from cluster averages. **f** ISH of *Ncr1* in endoneurium and epineurium areas of uninjured and injured (3, 12, and 60 days post-CCI) sciatic nerve. Asterisk: Necrotic area. Scale bars: 100 µm. **g** Bar chart with individual values displaying number of *Ncr1* + cells in uninjured (light gray), contralateral (blue), and ipsilateral (red, whole area) nerve for each timepoint (mean ± SD, *n* = 2–3 animals). **h** Percent *Ncr1* + cells in ISH data in (**f**) in epineurial and endoneurial areas. **i** ISH of *Cd3e* in endoneurium and epineurium areas of uninjured and injured (3, 12, and 60 days post-CCI) sciatic nerve. Asterisk: Necrotic area. Scale bars: 100 µm. **j** Bar chart with individual values displaying number of *Cd3e* + cells in uninjured (light gray), contralateral (blue), and ipsilateral (red, whole area) nerve for each timepoint (mean ± SD, *n* = 2–3 animals).

We finally subclustered lymphoid cells which resulted in 17 clusters (Fig. 7a, b and Supplementary Fig. 11). Previous studies of blood white cells have demonstrated the vast diversity and complex expression patterns that is present among this major cell group. In fact, several scOmics studies have demonstrated that combining transcriptomics and proteomics help annotate deeper subtypes among lymphoid subtypes^78^. In our study, we also observed widespread cluster heterogeneity and decided to not fully characterize clusters beyond major cell types. To this end, we identified eight different subtypes of T cells (*Cd3e*), five subtypes of NK/ILC1 cells (*Ncr1, Klrb1a*), ILC2 cells (*Pparg*), B cells (*Cd19*), and mast cells (*Cpa3*) (Fig. 7c–e)^55^. Interestingly, histological evaluation of the presence of *Cd3e* + T cells and *Ncr1* + NK cells revealed that in uninjured tissue these cells types were essentially absent (Fig. 7f–j), but that injury triggered NK cell infiltration primarily of the epineurial compartment, while T cells could be identified in both the epi- and endoneurial compartments.

### Potential paracrine interactions in anatomically restricted regions increase after injury

**Fig. 8:**
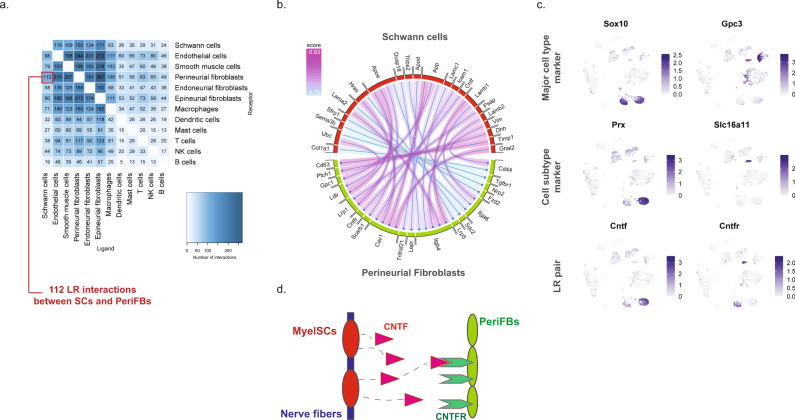
Ligand–receptor interactions in naive nerve. **a** Number of LR pairs between indicated cell types identified using SingleCellSignalR in naive nerve (LR > 0.05). **b** Circosplot of top interactions signaling from naive Schwann cells (ligand) to naive perineurial fibroblasts (receptor). **c** Feature plots validating cell type markers and identified LR pair in all naive cells. **d** Illustration of signaling between the LR pair, *Cntf*-*Cntfr*, between myelinating Schwann cells and perineurial fibroblasts. The LR score between myelinating Schwann cells and perineurial fibroblasts for *Cntf* and *Cntfr* is 0.84.

We next sought to explore the potential ligand–receptor (LR) interactions that exist between different cell types within naive nerve. We used a bioinformatic framework, SingleCellSignalR, to extract annotated LR pairs derived from a set of curated databases. Using this framework an LR score can be calculated and significant interactions can be identified based on scores between 0.5 and 1^79^. In naive nerve, we observed thousands of potential pairs among all cell types. Interestingly, the majority of LR pairs were between Schwann cells, fibroblasts, myeloid and vascular cells, whereas lymphoid cells occupied a much smaller fraction of potential pairs (Fig. 8a). Given no inflammatory response is anticipated in naive tissues, this observation further establishes confidence that the LR method provides physiologically relevant sensitivity. For exploring interactions between cell types, we focused on Schwann cell and perineurial fibroblasts which displayed anatomically proximity based on our histology data. We identified 112 potential interactions (LR score >0.5) from Schwann cells to perineurial fibroblasts. Among the top pairs (Fig. 8b), we identified the *Cntf-Cnftr* pair (LR score = 0.84) between myelinating Schwann cells and perineurial fibroblasts (Fig. 8c). *Cntf-Cntfr* has a known role in nerve development and inflammatory nociception, however, a direct interaction between myelinating Schwann cells and perineurial fibroblasts has not previously been demonstrated (Fig. 8d).

**Fig. 9:**
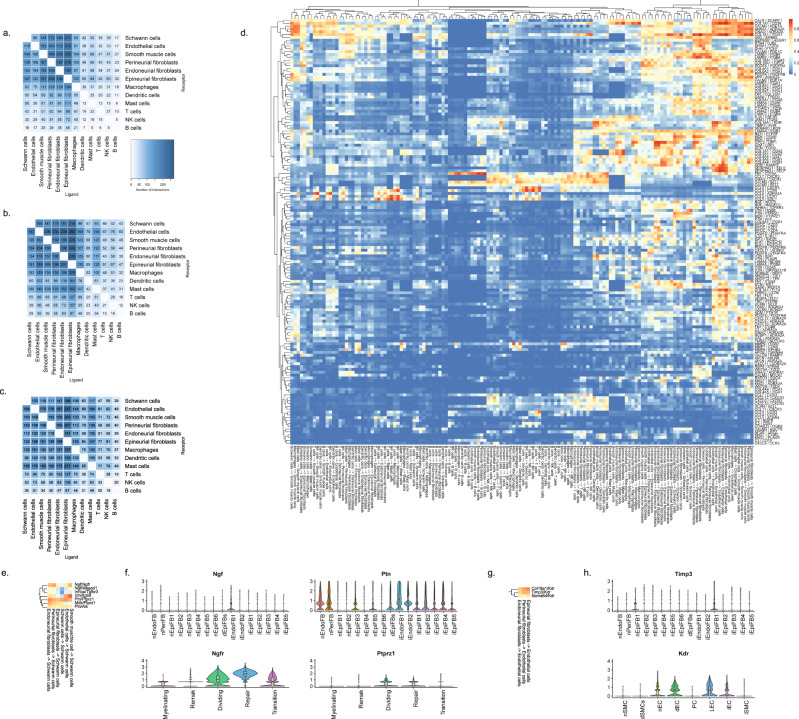
Ligand–receptor interactions after injury. Number of LR interactions larger than 0.5 between the indicated two cell types after **a** 3 days, **b** 12 days, and **c** 60 days of CCI injury, respectively. **d** Heat map of scaled expression of top 100 most-variable interactions at day 12. **e** Subset of interactions from (**d**) between types of fibroblasts and Schwann cells. **f** Violin plot with boxplot overlay and outliers (dots) of highlighted pairs, *Ngf - Ngfr* and *Ptn - Ptprz1*, from (**e**). **g** Subset of interactions from (**d**) between types of fibroblasts and vascular cells. **h** Violin plot with boxplot overlay and outliers (dots) of highlighted pair, *Timp3 - Kdr*, from (**g**).

When we explored the number of significant LR pairs after injury, we observed that the number increased especially at day 12 after injury. The distribution also changed with more interactions involving lymphoid and mast cells supporting the activation of an immune response (Fig. 9a–c). Exploring the top most-variable interactions at day 12 and focusing on genes that we identified in injury-specific clusters, we found that the activation of *Ngfr* in repair Schwann cells might derive from Ngf in endoneurial fibroblasts (Fig. 9d, f). In the same gene set, we identified another interaction between *Ptprz1* in repair cells and the ligand *Ptn* in almost all types of fibroblasts. We also identified a *Timp3-Kdr* (VEGFR) interaction between injured fibroblasts and endothelial cells suggesting an importance of fibroblasts in the negative regulation of angiogenesis after injury^80^. Among these LR pairs may be opportunities for pharmacological intervention for alleviating pain after nerve trauma.

## Discussion

In our study, we examined the molecular changes at single-cell resolution in uninjured naive nerve and in nerve after injury at three timepoints related to early (3 days) and late (12 days) responses to injury as well as at a more chronic state of tissue repair (60 days). At each of these timepoints, we were able to capture significant changes in fibroblasts, Schwann cells, and immune cells summing a total of 45 cell subtypes identified in naive nerve and 23 cell subtypes emerging after injury. Further, we identified numerous injury-specific ligand–receptor interactions that may be targeted for pharmacological intervention. While CCI nerve injury in rats is a widely used model in drug discovery for assessing the efficacy of analgesics in neuropathic pain assays, we are not able to conclude what genes are directly involved in the emergence or persistence of pain, or whether these targets translate to human pain conditions. This will require genetic or pharmacological validation of the targets in question and validation in human tissues, and remains a topic for future studies.

Peripheral nerve responds to injury with dramatic swelling and edema, in part due to increased vascularization, cell infiltration and proliferation. In this study, using histology we demonstrate that injury triggers a significant 3–6 fold increase in cell density compared to the contralateral side at 12 and 60 days. Investigation of proliferative markers in the scOmics data, including *Mki67* (Ki67), *Top2a*, and *Cdk1*^16^, indicate division among all major cell groups and reveal that Schwann cells and epineurial fibroblasts were the most proliferative types with increased densities ranging from 10 to 24 fold. We also identified a significant increase in the density of cell subtypes such as dendritic cells, T cells, and neutrophils which are commonly known to infiltrate tissues, further supporting that some of the increased density is due to infiltration. And last, histological analysis support increased vascularization in both the epi- and endoneurial compartment. Unexpectedly, we also identified a trend, although not significant, of increased cell density on the contralateral side after injury. This was consistent for the total nuclei density across all animals tested and peaked at day 3 or 12 before returning to uninjured levels at day 60.

Analysis of Schwann cells revealed that two subtypes, myelinated and unmyelinated Remak cells, are the prevailing types in naive nerve in agreement with the common consensus on glia in the periphery. In addition, we also identified that about 1% of naive Schwann cells are undergoing cell division suggesting naive nerve continue to replenish and repair even during homeostasis. After injury, however, cell division increased dramatically to 20%, and an injury-specific Schwann cell type emerged transiently and expressed markers of previously characterized Repair cells^19^. In fact, during the peak of inflammation, the majority of *Sox10* + Schwann were either dividing, transitional, or Repair cells, underscoring that the Schwann cell phenotype is highly plastic. Previous studies of Repair cells have suggested the potential of these cells to transition between myelinated and unmyelinated phenotypes via a Repair cell transition, although this has not been conclusively demonstrated^20^. In our study, we identified unique and selective markers of the Repair phenotype, which may aide future studies in interrogating the potential for targeting this injury-specific cell type for therapeutic purposes.

Here, we also identified the distinguishing molecular features of multiple subpopulations of myeloid cells in uninjured naive and injured sciatic nerve. We found eight distinct subtypes in naive nerve: *Cma1* + mast cells, *Xcr1*+/*Clec9a* + conventional self-renewing tissue-resident dendritic cells, five types of MHC II+ or C1q+ macrophages including one dividing and patrolling non-classical *N4a1r* + monocyte population. Injury was marked by a dramatic increase in the density of myeloid cells, both in the epineurial and endoneurial compartments. Our data further identified the emergence of injury-specific cell subtypes distinguished by molecular features and temporal patterning, and identified recruited neutrophils, *Siglech* + plasmacytoid dendritic cells, and nine injury subtypes of macrophages. A recent study by Ydens et al. report that the majority of endoneurial and epineurial macrophages are populated by late embryonic precursors that slowly replenish after birth by hematopoietic stem cells derived precursors suggesting this origin could remain in adulthood. This is supported by another study by Wang et al., who show by fate mapping, that the PNS is fully populated with macrophages at birth and only 8% of these derive from the yolk sac. This is in stark contrast to CNS microglia which derive almost exclusively from yolk sac progenitors. Using a Flt3 transgenic reporter line, Wang et al. further identify that 74% of nerve macrophages were derived from hematopoietic stem cell-derived progenitors. This is significantly more than what we find (~30% of nMP4 in naive nerve), but can be explained by differences in nerve harvest technique and how much epineurial tissue is included in the dissociate. While both reports identify two populations of macrophages distinguished by distinct markers (e.g., *Cd74*, MHC II genes) or (*Clec10a*, *Lyve1*^*Hi*^), Ydens et al. further show *Clec10a* is restricted to the epineurial compartment. This finding is supported by a recent study in lung tissues where MHC II genes and *Lyve1* distinguished macrophages associated with nerve bundles and vessels, respectively, adding further support to anatomical functional specialization in the peripheral nerve compartment^81^. Based on these previously validated markers, our study suggests that the epineurial compartment is populated by three subtypes of self-maintaining macrophages involved in tissue wound repair, while the endoneurial compartment is populated by one subtype of macrophage derived from both embryonic and hematopoietic stem cells lineages, but with a similar phenotype involved in antigen presentation. Further studies are needed to fully delineate the molecular types and their respective functional roles in homeostasis and repair.

In our study, fibroblasts had the most diverse phenotypical heterogeneity. We identified three different anatomical types comprising more than nine types in naive nerve and an additional seven types in injured nerve. Histological analysis demonstrated robust proliferation and the development of an inflammatory profile among fibroblasts as well as the emergence of many injury triggered epineurial types peaking at day 12. The role of fibroblasts in nociception remains not fully understood. A previous study by Carr et al. investigated nerve injury and proposed that endoneurial fibroblasts, also termed mesenchymal cells, possess precursor potential for bone and dermal repair^6^. The major cell type with the least heterogeneity were vascular cells. This cell group also displayed minimal emergence of distinct subtypes after injury although types displayed robust DEGs after, but not before, injury. This is not surprising since the vasculature may be uniquely equipped with mechanisms to quickly deal with neurogenic inflammation.

Lymphoid cells displayed a unique profile. While the uninjured nerve was essentially devoid of lymphoid cells, many different subtypes infiltrated after injury, which was also supported by histological evidence. Given the known challenges in phenotyping lymphoid cell subtypes based on RNA alone and without protein markers^78^, we decided not to pursue annotation of this cell group in detail. However, interestingly, histological evaluation of uninjured and injured nerve demonstrated that NK cells predominantly occupied the epineurial compartment while T cells occupied the endoneurial compartment.

Exploring ligand–receptor interactions revealed that injury caused an increase in the number and distribution of interactions, as observed in previous studies examining interactions between sensory neurons and other tissues or cell types^82^. Our analysis revealed the potential importance of fibroblast cells in all compartments of the nerve based on the identification of putative LR pairs to injury-specific Schwann cells as well as endothelial cells. Although our analysis did not investigate the molecular changes of DRG neurons after injury, previous studies of DRG have demonstrated that neurons adopt a different molecular profile after injury^13^. Future analysis integrating these studies and validating LR pairs may delineate a molecular sequence of changes and identification of the cellular origins. This in turn, may provide a path for the discovery of novel analgesics that prevent the development of persistent neuropathic pain after injury.

Altogether, our study provides insight into the vast heterogeneity among cell subtypes in different nerve compartments of the uninjured and injured nerve. Our goal with this study was to provide a descriptive foundation of cellular heterogeneity in naive and injured nerve. While our study provides sufficient resolution to identify many novel cell subtypes, to the best of our knowledge, among the 45 cell subtypes in naive nerve and 23 cell subtypes emerging after injury, there may be still subtypes that we our study could not resolve bioinformatically, but that require even larger Omics studies of nerve to resolve. However, while this report documents the emergence of cell subtypes, the function of many of these types still remains unclear and was not the scope of this study. We hope this work will enable future studies aimed at identifying cells of druggable interest for the prevention and symptomatic treatment of emergence and persistence of neuropathic pain.

## Methods

### CCI model generation, perfusion, and nerve extraction

Sprague Dawley rats weighed between 175 and 250 g at the time of surgery. The procedure was performed under aseptic conditions, and using standard sterile technique to perform CCI (or Sham-CCI) surgery. Anesthesia (3% Isoflurane in 100% Oxygen) was delivered by a nose cone, connected to an anesthetic machine containing a scavenging system. The skin over the back and left thigh was shaved and wiped alternately 3 times with alcohol and Betadine. The animals laid on a heating pad, temperature regulated. An incision was made to expose the left common sciatic nerve at mid-thigh level. Four loose ligatures (catgut chromic 4-0) were applied to the sciatic nerve (1 mm apart) in order to induce a local constriction injury according to the model by Bennett and Xie^10^. The skin was closed with wound clips. For all studies conducted at the Research Labs of Merck & Co., Inc., Kenilworth, NJ, USA, experimental procedures described herein were approved by the Institutional Animal Care and Use Committees, and subjects were housed and tested in AAALAC-accredited facilities.

### Enzymatic digestion and single-cell RNA-seq

About 10 mm of naive sciatic nerve or 10 mm of the CCI ligated sciatic nerve was dissected from rats transcardially perfused with PBS for immediate enzymatic dissociation. Nerve tissues were finely chopped with scalpels and then digested in 1–2 ml enzyme cocktail comprising: trypsin (Gibco, 3 mg/ml), collagenase II (Worthington 800 U/ml), hyaluronidase (Worthington, 1105 U/ml), pronase (Sigma, 240 U/ml), and dispase (Sigma, 5 U/ml) in HEPES buffered HBSS with 0.45% glucose. The digestion continued at 37 °C and 5% CO_2_, and was terminated after 40–60 min by adding 1–2 ml dissociation medium (HBSS with HEPES, 0.45% glucose, and 1% BSA) followed by trituration with a p1000 pipette. The dissociate was then filtered over a 40 µm filter and into a 50 ml tube on ice and 10 ml dissociation medium was added. Cells were centrifugated (10 min, 1000 rpm, 10 °C) and resuspended in 1 ml dissociation medium for an additional two washes. Cells were finally resuspended in PBS with 1% BSA and counted. Single-cell RNA-sequencing was performed using the Chromium Single Cell 3′ Kit with v2 chemistry (10X Genomics) according to manufacturer instructions. We targeted 8000 cells per 10X GEM reaction. Libraries were QC’ed by verifying optimal size using the High Sensitivity DNA Agilent Bioanalyzer kit and cDNA concentration quantified using the Invitrogen Qubit HS DNA kit according to the manufacturer’s instructions. Then libraries were sequenced on an Illumina Novaseq 6000.

### scRNA-seq data analysis

Raw fastq files were demultiplexed with CellRanger (v3.0.0) and aligned using the rat genome (Rn6). Downstream analyses were performed with R (v.3.5) and Seurat (v.3.1). Cells with 200-5,500 genes and <10% mitochondrial reads were retained. Data were normalized using sctransform and batch correction was performed using harmony. 30 principal components and resolution = 0.8 were used for dimensionality reduction and clustering, respectively. Clusters of red blood cells (high *Hbb*) and doublets/debris (multiple cell type markers, low gene counts) were removed and the data was reprocessed using the same parameters. Dendritic cells and B cells both expressed high MHC levels and were clustered together at resolution 0.8. Expression of *Cd19* and *Siglech* confirmed two distinct populations which were subsequently reclustered at resolution 0.1 to yield distinct populations. Major populations were subclustered using the same parameters. Detecting highly variable genes, finding clusters and creating UMAP dimplots, feature plots, violin plots, dot plots, and gene expression heatmaps plots was done using R (v.3.6) and Seurat (v.3.1). All violin plots are displayed with an overlayed boxplot where the lower, middle and upper hinges are the 25th percentile, median and 75th percentile, respectively, and the bottom and top whiskers are the minimum and maximum values. The gray circles in the boxplot display the outliers. Heatmaps of genes display scaled expression across each gene. Identification of marker genes in clusters was found using the FindAllMarkers function, and heatmaps showing average cluster expression were done using the AverageExpression function of the Seurat pipeline on SCT data. Heatmaps showing cell counts were produced using the table function in Seurat. To identify potential paracrine interactions we used SingleCellSignalR (v.1.0) in R (v.4.0) to mine 3251 LR pairs in an annotated dataset containing all cell types. SingleCellSignalR was used on an already annotated dataset exported from Seurat.

### RNAscope

RNA in situ hybridization experiments were performed using the RNAscope® technology on FFPE section from nerve of animals (*n* = 3 animals per condition, unless noted otherwise) in the same cohorts as those used for single-cell RNA-seq. Paired double-Z oligonucleotide probes were designed against the following target RNA from rat: Cd68 (NM_001031638.1), Cd3e (NM_001108140.1), Cdh5 (NM_001107407.1), Des (NM_022531.1), Sox10 (NM_019193.2), Sfrp2 (NM_001100700.1), Siglech (XM_008759473.2), Gpc3 (NM_012774.1), and Ncr1 (NM_057199.1). Hybridization was detected using the RNAscope 2.5 LS Reagent RED Kit (ACD) according to the manufacturer’s instructions where the gene of interest is stained red, and all nuclei are blue. Each sample was quality controlled for RNA integrity with a probe specific to the housekeeping genes POLAR2a, PPIB, and UBC. Negative control background staining was evaluated using a probe specific to the bacterial dapB gene. Brightfield images were acquired using a AperioXT microscope using a 40x objective and images were analyzed in HALO (v.3.2).

### Statistics and reproducibility

Differential gene expression was quantified using the Seurat pipeline using the FindAllMarkers function which identifies differentially expressed genes between two groups of cells using a Wilcoxon Rank Sum test. *P* values were filtered at *p* < 0.05 and returning only genes upregulated against the comparator (pos.only = TRUE)). Statistical analysis of imaging data quantified in HALO was performed in Graph Prism version 8 using a ANOVA analysis with multiple comparison test as indication in figure legend. Supplementary Table 1 outlines the biological and technical sample sizes of the scRNA-seq data. Supplementary Fig. 2 outlines the sample sizes of each annotated cell type per timepoint of the scRNA-seq data.

### Reporting summary

Further information on research design is available in the Nature Research Reporting Summary linked to this article.

## Supplementary information

Supplementary Information

Description of Additional Supplementary Files

Supplementary Data 1

Reporting Summary

## Data Availability

Source data is provided for Figs. 1f, g, 2i, 3h, k, 4g, 5c, d, 6b, 6j, 7g, h, and j in Supplementary Data 1. Single-cell RNA-seq data that support the findings of this study is deposited in GEO Omnibus with the accession code (http://www.ncbi.nlm.nih.gov/).

## References

[1] Binder A, Baron R (2016). The pharmacological therapy of chronic neuropathic pain. Dtsch. Arztebl. Int..

[2] Woolf CJ (2020). Capturing novel non-opioid pain targets. Biol. Psychiatry.

[3] Smith MT, Anand P, Rice ASC (2016). Selective small molecule angiotensin II type 2 receptor antagonists for neuropathic pain: preclinical and clinical studies. Pain.

[4] Shepherd AJ (2018). Macrophage angiotensin II type 2 receptor triggers neuropathic pain. Proc. Natl Acad. Sci. USA.

[5] Yuste R (2020). A community-based transcriptomics classification and nomenclature of neocortical cell types. Nat. Neurosci..

[6] Carr MJ (2019). Mesenchymal precursor cells in adult nerves contribute to mammalian tissue repair and regeneration. Cell Stem Cell.

[7] Wolbert J (2020). Redefining the heterogeneity of peripheral nerve cells in health and autoimmunity. Proc. Natl Acad. Sci. USA.

[8] Wang PL (2020). Peripheral nerve resident macrophages share tissue-specific programming and features of activated microglia. Nat. Commun..

[9] Ydens E (2020). Profiling peripheral nerve macrophages reveals two macrophage subsets with distinct localization, transcriptome and response to injury. Nat. Neurosci..

[10] Bennett GJ, Xie YK (1988). A peripheral mononeuropathy in rat that produces disorders of pain sensation like those seen in man. Pain.

[11] Chen H (2018). Effect of autophagy on allodynia, hyperalgesia and astrocyte activation in a rat model of neuropathic pain. Int. J. Mol. Med..

[12] Chen JY (2018). Valproate reduces neuroinflammation and neuronal death in a rat chronic constriction injury model. Sci. Rep..

[13] Renthal W (2020). Transcriptional reprogramming of distinct peripheral sensory neuron subtypes after axonal injury. Neuron.

[14] Gillespie CS, Sherman DL, Blair GE, Brophy PJ (1994). Periaxin, a novel protein of myelinating Schwann cells with a possible role in axonal ensheathment. Neuron.

[15] Yang Z, Wang KK (2015). Glial fibrillary acidic protein: from intermediate filament assembly and gliosis to neurobiomarker. Trends Neurosci..

[16] Elmentaite R (2021). Cells of the human intestinal tract mapped across space and time. Nature.

[17] Nocera G, Jacob C (2020). Mechanisms of Schwann cell plasticity involved in peripheral nerve repair after injury. Cell. Mol. Life Sci..

[18] Watanabe E, Hiyama TY, Kodama R, Noda M (2002). NaX sodium channel is expressed in non-myelinating Schwann cells and alveolar type II cells in mice. Neurosci. Lett..

[19] Jessen, K. R. & Mirsky, R. The success and failure of the Schwann cell response to nerve injury. *Front. Cell. Neurosci.* **13**, 10.3389/fncel.2019.00033 (2019). 10.3389/fncel.2019.00033 PMC6378273 30804758

[20] Jessen KR, Mirsky R (2016). The repair Schwann cell and its function in regenerating nerves. J. Physiol..

[21] Uchida H, Nagai J, Ueda H (2014). Lysophosphatidic acid and its receptors LPA1 and LPA3 mediate paclitaxel-induced neuropathic pain in mice. Mol. Pain..

[22] Yang N (2013). Generation of oligodendroglial cells by direct lineage conversion. Nat. Biotechnol..

[23] Marcinkiewicz M, Savaria D, Marcinkiewicz J (1998). The pro-protein convertase PC1 is induced in the transected sciatic nerve and is present in cultured Schwann cells: comparison with PC5, furin and PC7, implication in pro-BDNF processing. Mol. Brain Res..

[24] Gomes I (2016). Identification of GPR83 as the receptor for the neuroendocrine peptide PEN. Sci. Signal..

[25] Mack SM, Gomes I, Devi LA (2019). Neuropeptide PEN and its receptor GPR83: distribution, signaling, and regulation. ACS Chem. Neurosci..

[26] Torii T (2014). In vivo knockdown of ErbB3 in mice inhibits Schwann cell precursor migration. Biochem. Biophys. Res. Commun..

[27] Tabib T, Morse C, Wang T, Chen W, Lafyatis R (2018). SFRP2/DPP4 and FMO1/LSP1 define major fibroblast populations in human skin. J. Investigative Dermatol..

[28] Siu, M. K. Y. et al. Hexokinase 2 regulates ovarian cancer cell migration, invasion and stemness via FAK/ERK1/2/MMP9/NANOG/SOX9 signaling cascades. *Cancers* **11**, 10.3390/cancers11060813 (2019). 10.3390/cancers11060813 PMC6627345 31212816

[29] Ma W, Chabot JG, Vercauteren F, Quirion R (2010). Injured nerve-derived COX2/PGE2 contributes to the maintenance of neuropathic pain in aged rats. Neurobiol. Aging.

[30] Zhuang GZ (2015). Carbonic anhydrase-8 regulates inflammatory pain by inhibiting the ITPR1-cytosolic free calcium pathway. PLoS ONE.

[31] Zhuang GZ (2018). Human carbonic anhydrase-8 AAV8 gene therapy inhibits nerve growth factor signaling producing prolonged analgesia and anti-hyperalgesia in mice. Gene Ther..

[32] Sawant KV (2016). Chemokine CXCL1 mediated neutrophil recruitment: role of glycosaminoglycan interactions. Sci. Rep..

[33] Venkatesh D (2013). Endothelial TNF receptor 2 induces IRF1 transcription factor-dependent interferon-β autocrine signaling to promote monocyte recruitment. Immunity.

[34] Yang X (2010). Conditional expression of Spry1 in neural crest causes craniofacial and cardiac defects. BMC Dev. Biol..

[35] Ben-Kraiem A (2021). Selective blood-nerve barrier leakiness with claudin-1 and vessel-associated macrophage loss in diabetic polyneuropathy. J. Mol. Med..

[36] Li S (2015). GDF10 is a signal for axonal sprouting and functional recovery after stroke. Nat. Neurosci..

[37] Farrukh F, Davies E, Berry M, Logan A, Ahmed Z (2019). BMP4/Smad1 signalling promotes spinal dorsal column axon regeneration and functional recovery after injury. Mol. Neurobiol..

[38] Heinke J (2008). BMPER is an endothelial cell regulator and controls bone morphogenetic protein-4-dependent angiogenesis. Circulation Res..

[39] Nomura S (2008). FGF10/FGFR2 signal induces cell migration and invasion in pancreatic cancer. Br. J. Cancer.

[40] Tong L (2016). Fibroblast growth factor-10 (FGF-10) mobilizes lung-resident mesenchymal stem cells and protects against acute lung injury. Sci. Rep..

[41] English AW, Liu K, Nicolini JM, Mulligan AM, Ye K (2013). Small-molecule trkB agonists promote axon regeneration in cut peripheral nerves. Proc. Natl Acad. Sci. USA.

[42] Rao J-S (2018). NT3-chitosan enables de novo regeneration and functional recovery in monkeys after spinal cord injury. Proc. Natl Acad. Sci. USA.

[43] Muhl L (2020). Single-cell analysis uncovers fibroblast heterogeneity and criteria for fibroblast and mural cell identification and discrimination. Nat. Commun..

[44] Karnik SK (2003). A critical role for elastin signaling in vascular morphogenesis and disease. Development.

[45] Schweitzer, K. S. et al. IGSF3 mutation identified in patient with severe COPD alters cell function and motility. *JCI Insight* **5**, 10.1172/jci.insight.138101 (2020). 10.1172/jci.insight.138101 PMC7453886 32573489

[46] Zheng L (2022). Telmisartan relieves liver fibrosis and portal hypertension by improving vascular remodeling and sinusoidal dysfunction. Eur. J. Pharm..

[47] Kang X (2020). Neuropeptide Y acts directly on cartilage homeostasis and exacerbates progression of osteoarthritis through NPY2R. J. Bone Min. Res..

[48] Obata K (2016). Tachykinin receptor 3 distribution in human oral squamous cell carcinoma. Anticancer Res..

[49] Mackay F, Browning JL (2002). BAFF: a fundamental survival factor for B cells. Nat. Rev. Immunol..

[50] Janssens R, Struyf S, Proost P (2018). The unique structural and functional features of CXCL12. Cell. Mol. Immunol..

[51] Kwiatkowski K (2019). Chemokines CCL2 and CCL7, but not CCL12, play a significant role in the development of pain-related behavior and opioid-induced analgesia. Cytokine.

[52] van Nieuwenhoven FA (2017). Cartilage intermediate layer protein 1 (CILP1): a novel mediator of cardiac extracellular matrix remodelling. Sci. Rep..

[53] Cancel, J.-C., Crozat, K., Dalod, M. & Mattiuz, R. Are conventional type 1 dendritic cells critical for protective antitumor immunity and how? *Front. Immunol.* **10**, 10.3389/fimmu.2019.00009 (2019). 10.3389/fimmu.2019.00009 PMC6379659 30809220

[54] Wohn C (2020). Absence of MHC class II on cDC1 dendritic cells triggers fatal autoimmunity to a cross-presented self-antigen. Sci. Immunol..

[55] Villani A-C (2017). Single-cell RNA-seq reveals new types of human blood dendritic cells, monocytes, and progenitors. Science.

[56] Wernersson S, Pejler G (2014). Mast cell secretory granules: armed for battle. Nat. Rev. Immunol..

[57] Caughey GH (2007). Mast cell tryptases and chymases in inflammation and host defense. Immunol. Rev..

[58] Carlin LM (2013). Nr4a1-dependent Ly6Clow monocytes monitor endothelial cells and orchestrate their disposal. Cell.

[59] Buscher, K., Marcovecchio, P., Hedrick, C. C. & Ley, K. Patrolling mechanics of non-classical monocytes in vascular inflammation. *Front. Cardiovasc. Med.* **4**, 10.3389/fcvm.2017.00080 (2017). 10.3389/fcvm.2017.00080 PMC5742122 29312957

[60] Rőszer T (2015). Understanding the mysterious M2 macrophage through activation markers and effector mechanisms. Mediators Inflamm..

[61] Bohlson, S. S., O’Conner, S. D., Hulsebus, H. J., Ho, M.-M. & Fraser, D. A. Complement, C1q, and C1q-related molecules regulate macrophage polarization. *Front. Immunol.* **5**, 10.3389/fimmu.2014.00402 (2014). 10.3389/fimmu.2014.00402 PMC4139736 25191325

[62] Chávez-Galán, L., Olleros, M. L., Vesin, D. & Garcia, I. Much more than M1 and M2 macrophages, there are also CD169+ and TCR+ macrophages. *Front. Immunol.* **6**, 10.3389/fimmu.2015.00263 (2015). 10.3389/fimmu.2015.00263 PMC4443739 26074923

[63] Diamond AG, Hood LE, Howard JC, Windle M, Winoto A (1989). The class II genes of the rat MHC. J. Immunol..

[64] Blasius AL, Colonna M (2006). Sampling and signaling in plasmacytoid dendritic cells: the potential roles of Siglec-H. Trends Immunol..

[65] Pruenster M (2015). Extracellular MRP8/14 is a regulator of β2 integrin-dependent neutrophil slow rolling and adhesion. Nat. Commun..

[66] Wache C (2015). Myeloid-related protein 14 promotes inflammation and injury in meningitis. J. Infect. Dis..

[67] Soehnlein O, Lindbom L (2010). Phagocyte partnership during the onset and resolution of inflammation. Nat. Rev. Immunol..

[68] Reichel CA (2009). Ccl2 and Ccl3 mediate neutrophil recruitment via induction of protein synthesis and generation of lipid mediators. Arteriosclerosis, Thrombosis, Vasc. Biol..

[69] Li JL (2016). Neutrophils self-regulate immune complex-mediated cutaneous inflammation through CXCL2. J. Investigative Dermatol..

[70] Kolaczkowska E, Kubes P (2013). Neutrophil recruitment and function in health and inflammation. Nat. Rev. Immunol..

[71] Shi C, Pamer EG (2011). Monocyte recruitment during infection and inflammation. Nat. Rev. Immunol..

[72] Lindborg JA, Mack M, Zigmond RE (2017). Neutrophils are critical for myelin removal in a peripheral nerve injury model of Wallerian degeneration. J. Neurosci..

[73] Vanlandewijck M (2018). A molecular atlas of cell types and zonation in the brain vasculature. Nature.

[74] Astarita, J., Acton, S. & Turley, S. Podoplanin: emerging functions in development, the immune system, and cancer. *Front. Immunol.* **3**, 10.3389/fimmu.2012.00283 (2012). 10.3389/fimmu.2012.00283 PMC3439854 22988448

[75] Vaahtomeri K (2017). Locally triggered release of the chemokine CCL21 promotes dendritic cell transmigration across lymphatic endothelia. Cell Rep..

[76] Wigle JT (2002). An essential role for Prox1 in the induction of the lymphatic endothelial cell phenotype. EMBO J..

[77] Fensterl V, Wetzel JL, Sen GC, Lyles DS (2014). Interferon-induced protein Ifit2 protects mice from infection of the peripheral nervous system by vesicular stomatitis virus. J. Virol..

[78] Hao Y (2021). Integrated analysis of multimodal single-cell data. Cell.

[79] Cabello-Aguilar S (2020). SingleCellSignalR: inference of intercellular networks from single-cell transcriptomics. Nucleic Acids Res..

[80] Qi JH (2003). A novel function for tissue inhibitor of metalloproteinases-3 (TIMP3): inhibition of angiogenesis by blockage of VEGF binding to VEGF receptor-2. Nat. Med..

[81] Chakarov S (2019). Two distinct interstitial macrophage populations coexist across tissues in specific subtissular niches. Science.

[82] Wangzhou A (2021). A ligand-receptor interactome platform for discovery of pain mechanisms and therapeutic targets. Sci. Signal..

