# Supplementary material for: scRNA-seq generates a molecular map of emerging cell subtypes after sciatic nerve injury in rats
Source: Commun Biol. 2022 Oct 19;5:1105. doi: 10.1038/s42003-022-03970-0 (PMC9581950; doi:10.1038/s42003-022-03970-0)
Supplement: Supplementary file 1 — Supplementary Information [file 42003_2022_3970_MOESM1_ESM.pdf]

Supplementary Information

scRNA-Seq Generates A Molecular Map Of Emerging Cell Subtypes After Sciatic Nerve Injury In Rats

Ditte Lovatt[1]*, Alex Tamburino[2], Alicja Krasowska-Zoladek[1], Raul Sanoja[1,4], Lixia Li[3], Vanessa Peterson[3], Xiaohai Wang[1], and Jason Uslaner[1]

[1]Department of Neuroscience, Merck & Co., Inc., West Point, PA, USA

[2]Department of Data and Genome Sciences, Merck & Co., Inc., West Point, PA, USA

[3]Department of Genome and Biomarker Science, Merck & Co., Inc., Boston, MA, USA

[4]New address: Biomarkers & Imaging, Vertex Pharmaceuticals, Boston, MA, USA

*Corresponding author: Ditte.Lovatt@merck.com

- Supplementary Table 1
- Supplementary Figures 1-11

Supplementary Table 1

CCI day	Library ID	Animal # (Biological replicate)	Technical sequencing replicate	Total cells
naïve	B5S22a_Naïve	22	a	1709
naïve	B5S22b_Naïve	22	b	2225
naïve	B5S23b_Naïve	23	b	2513
naïve	B5S24a_Naïve	24	a	1496
naïve	B5S24b_Naïve	24	b	2408
Day 3	B5S1a_CCId3	1	a	2429
Day 3	B5S1b_CCId3	1	b	2446
Day 3	B5S4a_CCId3	4	a	1570
Day 3	B5S4b_CCId3	4	b	1611
Day 3	B5S5a_CCId3	5	a	1295
Day 3	B5S5b_CCId3	5	b	1477
Day 3	B5S6a_CCId3	6	a	4435
Day 3	B5S6b_CCId3	6	b	2954
Day 12	B4S1_CCId12	1	a	5523
Day 12	B5S3_CCId12	3	a	5846
Day 12	B5S2a_CCId12	2	a	5328
Day 12	B4S2b_CCId12	2	b	5234
Day 12	B4S4a_CCId12	4	a	5332
Day 12	B4S4b_CCId12	4	b	5069
Day 12	B4S5a_CCId12	5	a	5184
Day 12	B4S5b_CCId12	5	b	5142
Day 12	B4S6a_CCId12	6	a	4807
Day 12	B4S6b_CCId12	6	b	4551
Day 12	B4S7a_CCId12	7	a	5358
Day 12	B4S7b_CCId12	7	b	4727
Day 60	B6S31a_CCId60	31	a	4657
Day 60	B6S31b_CCId60	31	b	3419
Day 60	B6S32a_CCId60	32	a	3527
Day 60	B6S32b_CCId60	32	b	3848
Day 60	B6S33a_CCId60	33	a	4671
Day 60	B6S33b_CCId60	33	b	4730
Day 60	B6S34a_CCId60	34	a	2751
Day 60	B6S34b_CCId60	34	b	3249

Supplementary figure 1

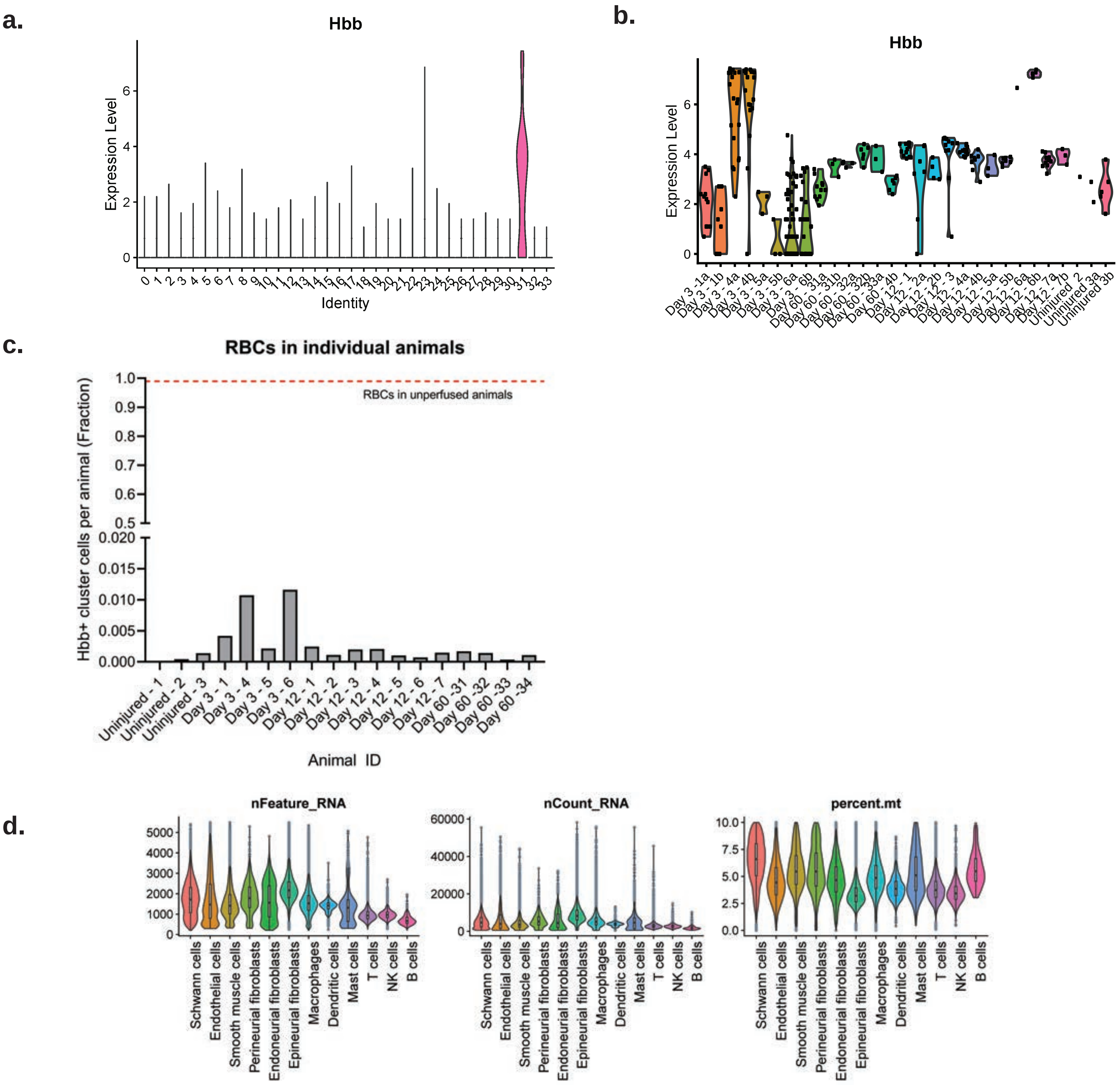

Supplementary Figure 1. Quality control of single-cell RNA-seq data. In pilot studies optimizing the nerve dissociation procedures, we observed that more than 90% of the recovered cells were red blood cells. This number was reduced to insignificant levels by transcardial perfusion of the rodent prior to nerve harvesting. **a)** To remove red blood cells, we perfused animals prior to nerve harvest. In the first iteration Seurat object of the normalized and clustered single-cell data (116,907 cells across 33 libraries), we detected a small Hbb positive cluster (Cluster 31, 298 cells). **b)** Almost all cells in cluster 31 expressed high levels of Hbb, establishing confidence that this cluster was primarily red blood cells. **c)** Exploring the contribution of cluster 31 Hbb+ cells in each animal established that perfusion remove the vast majority of red blood cells, and that less than 1% remained. **d)** Violin plots of the number of genes (nFeature_RNA), number of transcripts (nCount_RNA) and percent mitochondrial RNA (percent.mt).

Supplementary figure 2

Annotation of all cell groups/types

a.

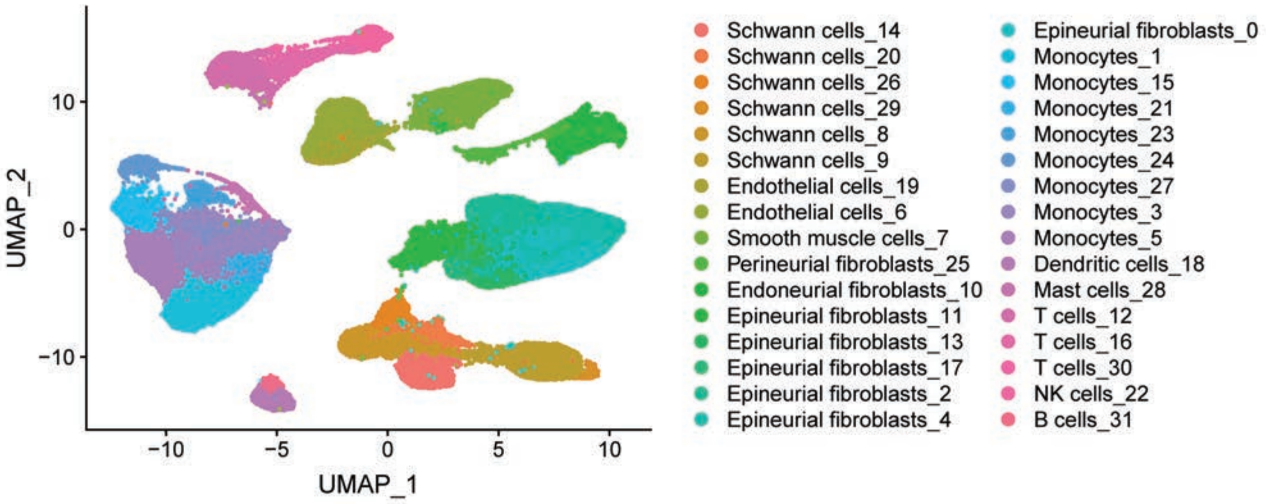

b.

Percentage per timepoint (Mean±STDEV)	Uninjured Naïve	Day 3	Day 12	Day 60
B cells_31	0.17±0.06	0.36±0.01	0.19±0	0.25±0.03
Dendritic cells_18	0.09±0.02	2.34±0.13	1.86±0	0.72±0.05
Endoneurial fibroblasts_10	8.83±0.69	6.99±0.36	3.77±0.01	2.1±0.2
Endothelial cells_19	2.9±0.3	0.76±0.09	1.61±0	1.97±0.06
Endothelial cells_6	15.05±0.54	5.81±0.42	3.04±0.01	5.56±0.07
Epineurial fibroblasts_0	1.5±0.29	5.27±0.66	10.95±0.02	10.88±0.38
Epineurial fibroblasts_11	1.12±0.09	8.94±0.86	4.32±0.01	2.41±0.12
Epineurial fibroblasts_13	0.44±0.11	0.65±0.04	4.05±0.01	4.06±0.19
Epineurial fibroblasts_17	1.8±0.17	0.14±0.01	2.23±0	1.94±0.17
Epineurial fibroblasts_2	2.79±0.17	1.74±0.18	5.82±0.01	14.3±0.44
Epineurial fibroblasts_4	1.4±0.18	3.16±0.4	5.5±0.01	10.09±0.59
Mast cells_28	0.1±0.01	1.09±0.04	0.41±0	0.48±0.02
Macrophages_1	2.96±0.14	9.43±0.98	7.17±0.01	8.5±0.3
Macrophages_15	2.27±0.23	2.76±0.21	2.71±0	2.67±0.04
Macrophages_21	0.12±0.01	1.89±0.07	2.08±0	0.79±0.06
Macrophages_23	1.25±0.19	1.63±0.08	1.34±0	1.75±0.07
Macrophages_24	0.31±0.04	2.45±0.17	1.29±0	0.56±0.02
Macrophages_27	0.11±0.02	1.02±0.06	0.59±0	0.28±0.02
Macrophages_3	1.16±0.07	14.06±0.47	5.38±0.01	6.1±0.27
Macrophages_5	3.21±0.24	5.06±0.55	6.36±0.01	6.33±0.39
NK cells_22	0.27±0.03	1.03±0.06	1.92±0	1.48±0.05
Perineurial fibroblasts_25	2.76±0.31	0.6±0.03	0.94±0	0.93±0.16
Schwann cells_14	11.77±0.98	0.59±0.01	3.16±0.01	0.97±0.05
Schwann cells_20	0.32±0.02	2.73±0.11	1.35±0	1.85±0.1
Schwann cells_26	0.25±0.03	2.94±0.16	0.84±0	0.13±0.01
Schwann cells_29	0±0	0±0	0.54±0	0±0
Schwann cells_8	0.3±0.05	3.96±0.18	7.24±0.01	1.1±0.03
Schwann cells_9	17±0.85	2.22±0.1	2.7±0	4.44±0.22
Smooth muscle cells_7	18.16±0.25	6.61±0.64	3.85±0.01	2.16±0.08
T cells_12	1.27±0.07	2.45±0.11	4.01±0.01	2.97±0.13
T cells_16	0.31±0.02	1.02±0.04	2.59±0	2.07±0.1
T cells_30	0.05±0	0.29±0.02	0.17±0	0.15±0.01
All cells	100	100	100	100

Supplementary Figure 2. Annotation and distribution of cells prior merging clusters. (A) UMAP plot of obtained clusters at utilized resolution as well as their annotation as indicated in the legend prior to merging clusters for figure 1 in the main manuscript. **(B)** Figure summarizing the percentage of cells in the indicated cluster across biological replicates per timepoint (mean +/- SD).

Supplementary figure 3

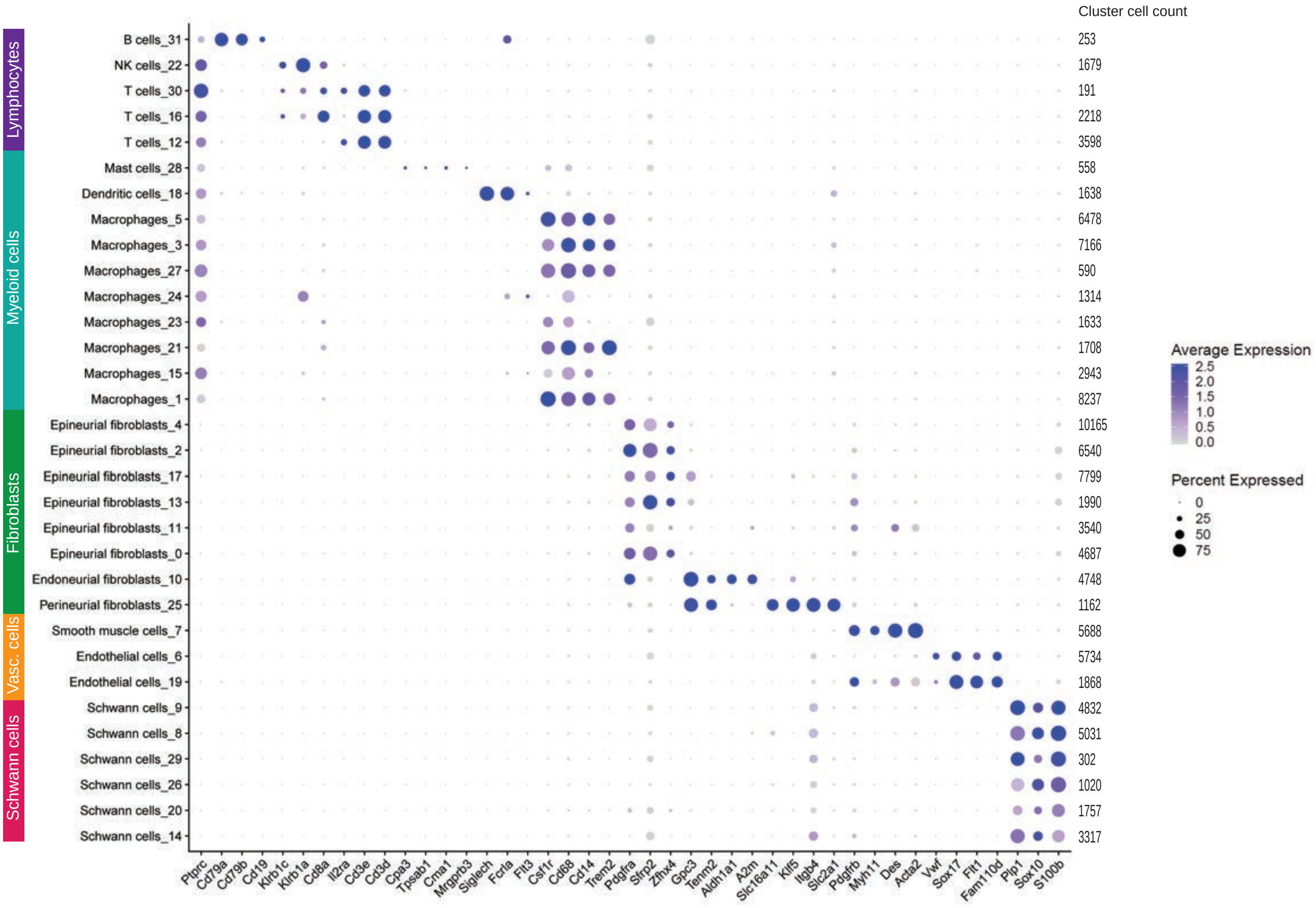

Supplementary Figure 3. Dot plot supporting the purity and merger of individual cluster into major cell groups/types in the final iteration of Seurat object of all timepoints based on the expression known markers. Number in the right column indicates number of cell count in the indicated cluster.

Supplementary figure 4

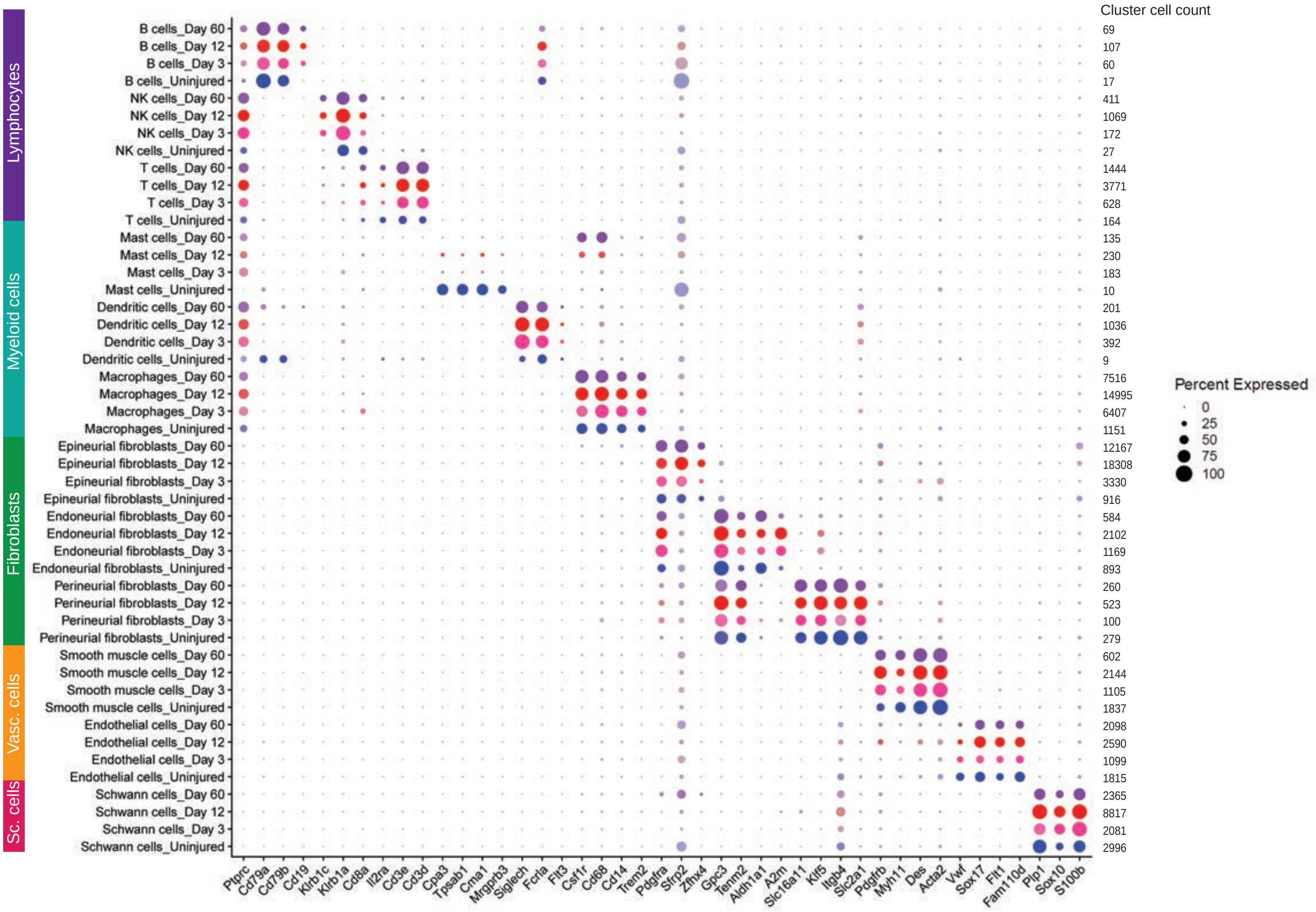

Supplementary Figure 4. Dot plot exploring the scaled expression of known markers across different timepoints in the final iteration of Seurat object after merging major cell groups/types. Blue, Uninjured; Pink, Day 3; Red, Day 12; Purple, Day 60. Number in the right column indicates the cell count in the indicated cluster.

Supplementary figure 5

Schwann cell subclustering

a.

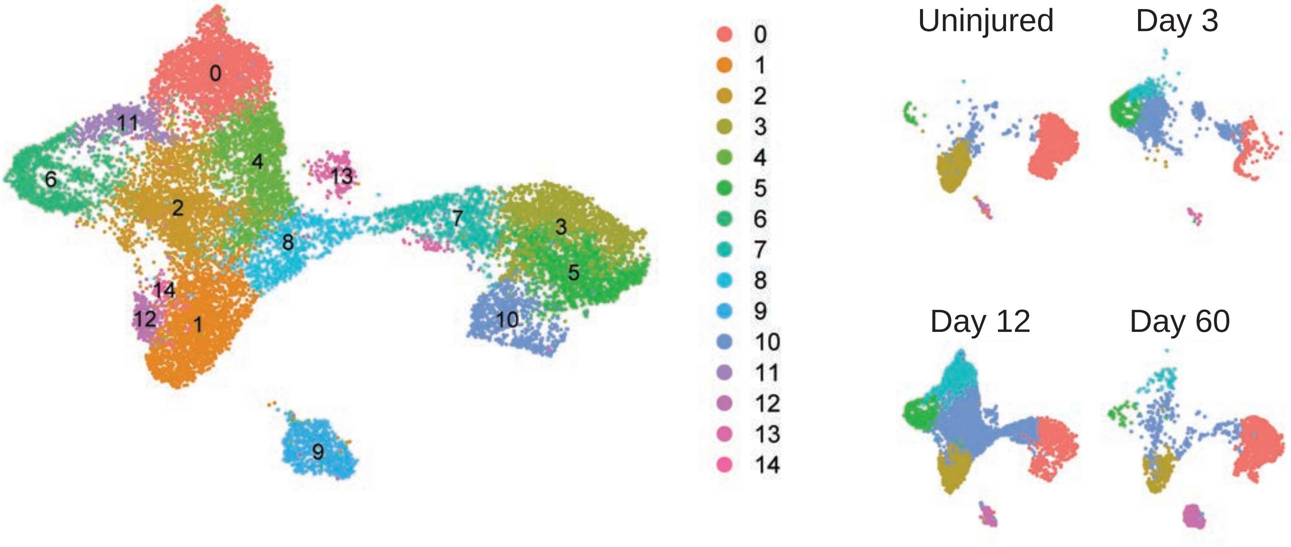

b.

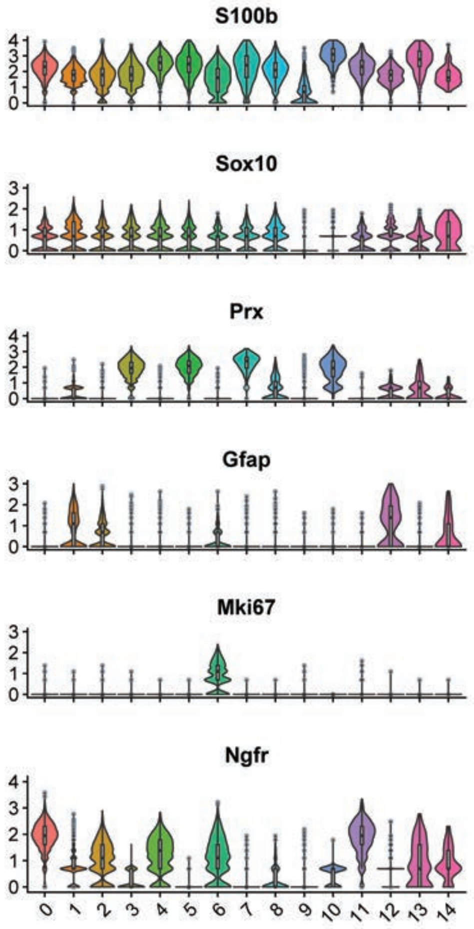

c.

Cluster ID	Cell subtype
0	Repair
1	Remak
2	Transition
3	Myelinating
4	Transition
5	Myelinating
6	Dividing
7	Transition
8	Transition
9	Unknown
10	Myelinating
11	Repair
12	Remak
13	Transition
14	Remak

Supplementary Figure 5. Subclustering on Glial and Schwann cell clusters (**a**). Annotation of Schwann cell types based on known markers (**b**) before merging of subclusters (**c**).

Supplementary figure 6

Naive remak and myelinating Schwann cell markers

a.

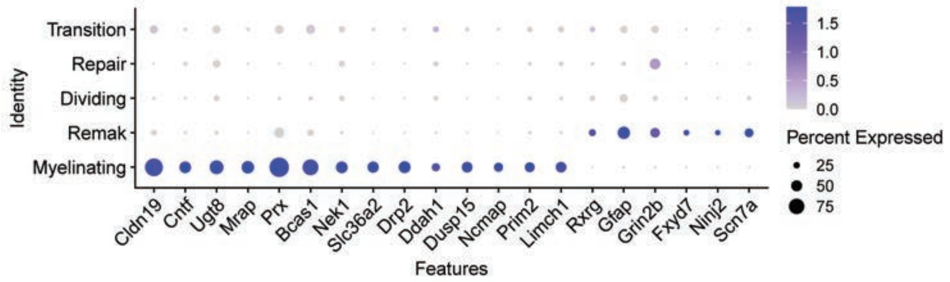

b.

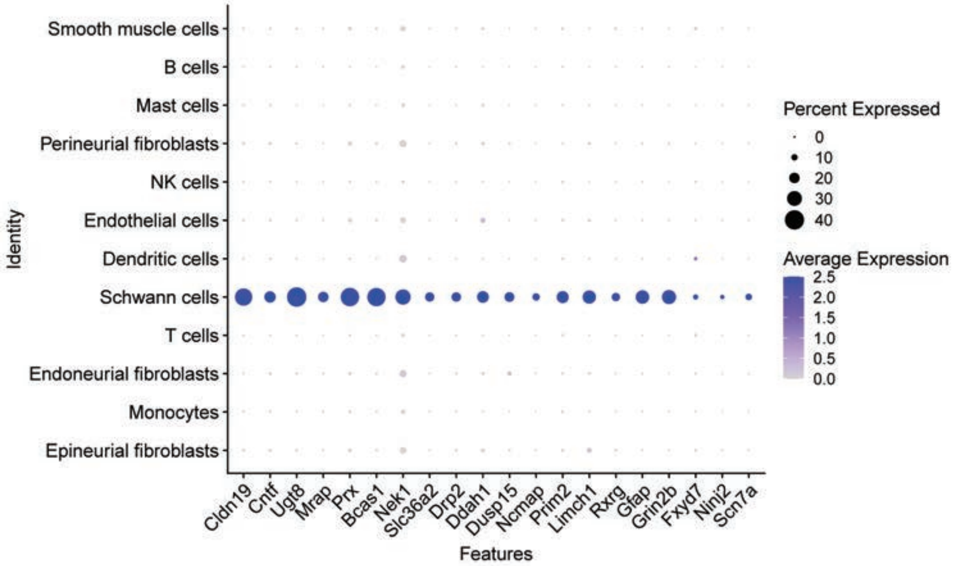

Supplementary Figure 6. Dot plots of scaled expression of genes. Validation of markers distinguishing Remak and Myelinating cells in naïve nerve (**a**) and distinguishing these cells from any of other cluster in nerve (**b**).

Supplementary figure 7

Injury specific Repair cell markers

a.

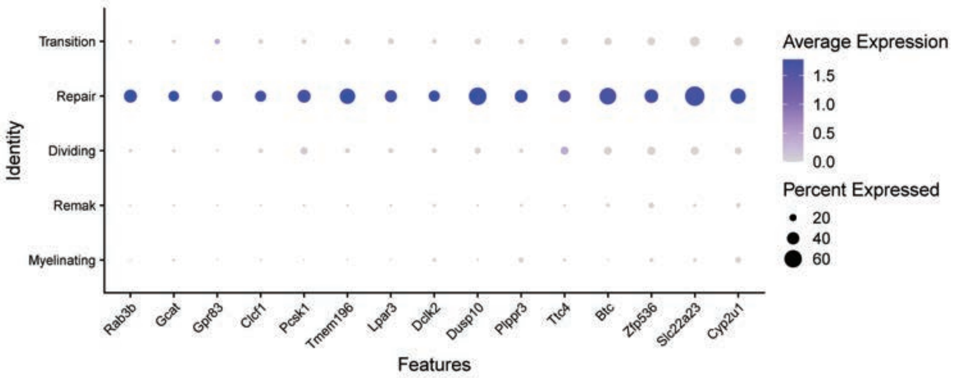

b.

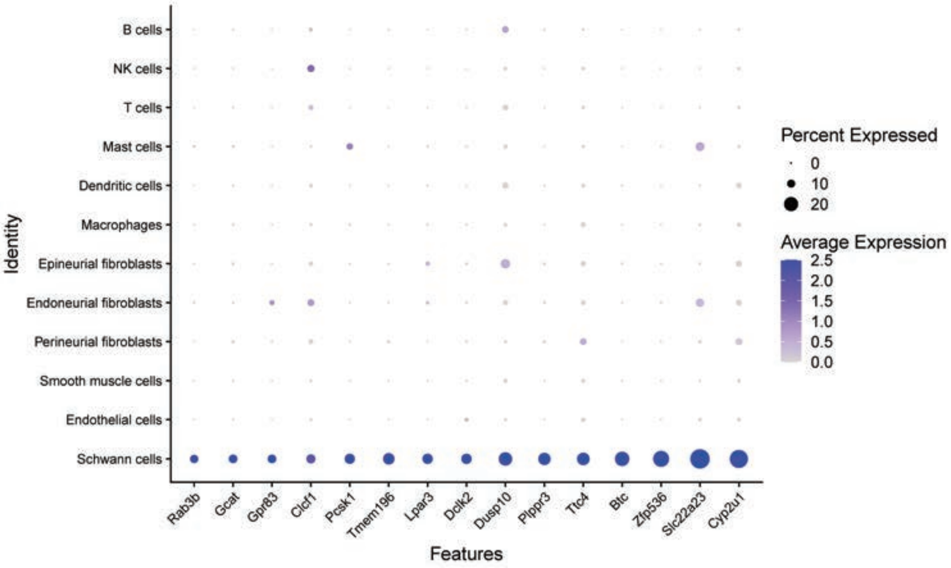

Supplementary Figure 7. Dot plots of scaled expression of genes. Validation of markers distinguishing Repair cells from other Schwann cell types in naïve nerve, including Remak and Myelinating cells (**a**). These markers are distinguishing repair cells from any of other cluster in nerve (**b**).

Supplementary Figure 8

Fibroblast annotation

a.

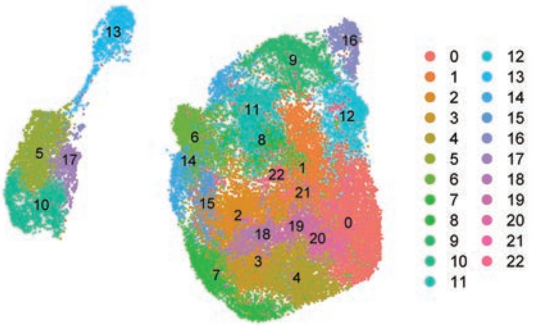

b.

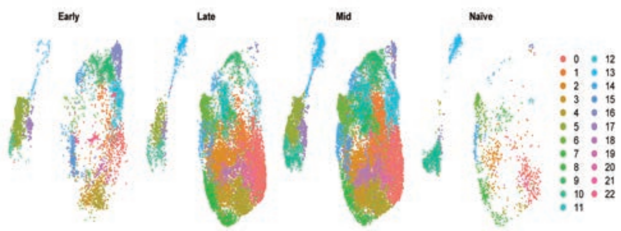

c.

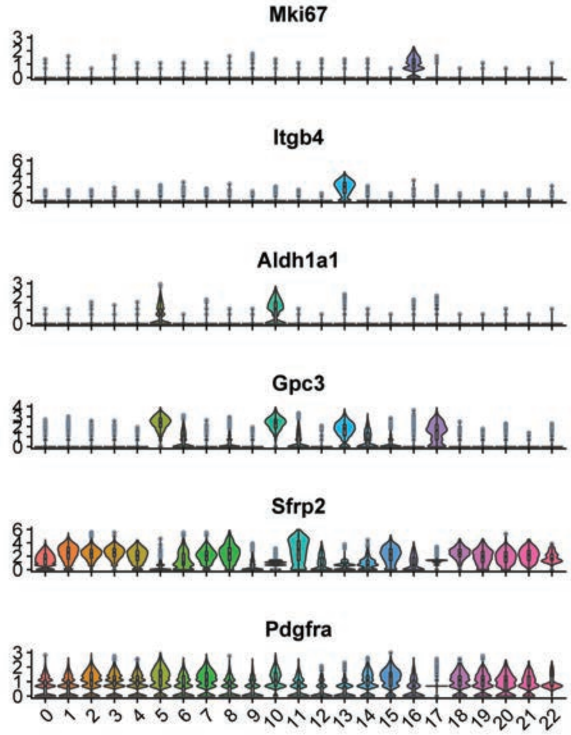

d.

Cluster ID	Cell subtype
0	nEpiFB1
1	iEpiFB4
2	nEpiFB5
3	iEpiFB3
4	iEpiFB3
5	iEndoFB1
6	nEpiFB4
7	nEpiFB2
8	nEpiFB6
9	iEpiFB1
10	nEndoFB
11	iEpiFB5
12	iEpiFB1
13	nPeriFB
14	nEpiFB3
15	iEpiFB2
16	dEpiFBs
17	iEndoFB2
18	iEpiFB3
19	iEpiFB3
20	nEpiFB1
21	iEpiFB4
22	iEpiFB4

e.

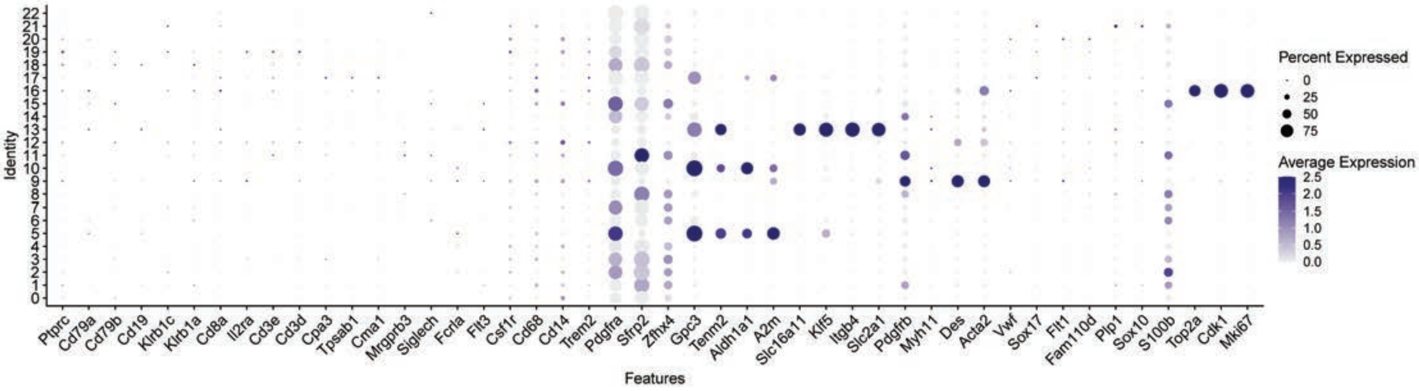

Supplementary Figure 8. Subclustering on Fibroblast cell clusters, at all timepoints (**a**) and at individual timepoints (**b**). Annotation of fibroblast cell types based on known markers (**c**) before merging of subclusters (**d-e**).(**e**) Dot plot of scaled expression of major cell type marker genes.

Supplementary Figure 9

Myeloid subcluster

a.

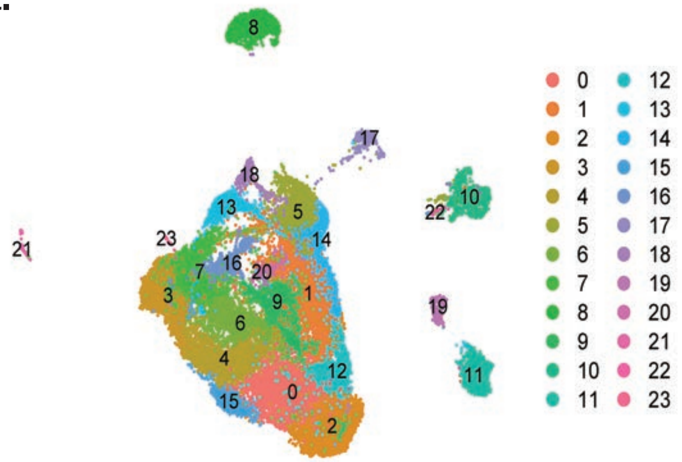

b.

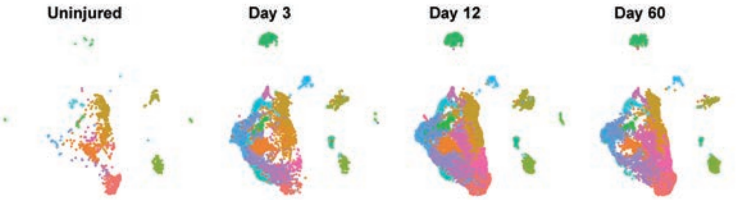

c.

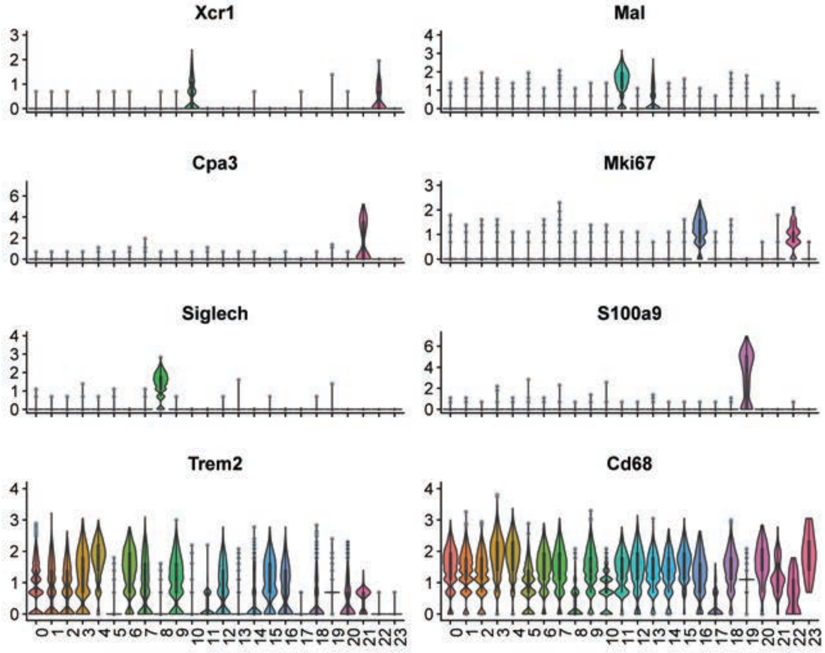

d.

Cluster ID	Cell subtype
0	iMP7
1	nMP3
2	nMP1
3	iMP4
4	iMP6
5	nMP4
6	nMP2
7	iMP5
8	pDC
9	iMP9
10	cDC1
11	PaMo
12	iMP10
13	iMP2
14	nMP4
15	iMP1
16	dMPs
17	iMP3
18	iMP8
19	NPs
20	iMP9
21	MCs
22	cDC1
23	Unknown

e.

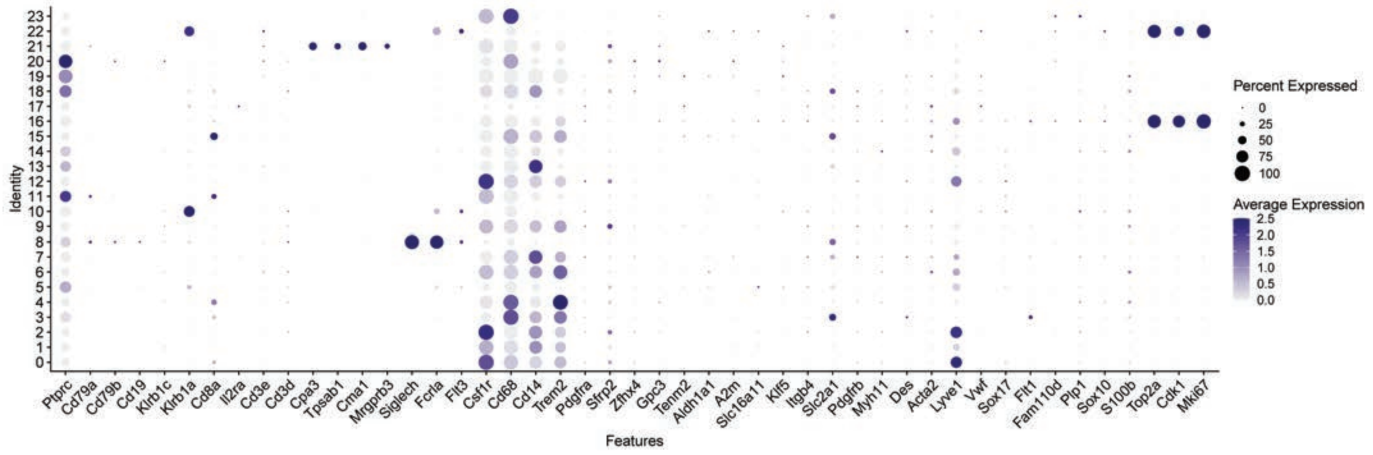

Supplementary Figure 9. Subclustering on Myeloid cell clusters, at all timepoints (**a**) and at individual timepoints (**b**). Annotation of Myeloid cell types based on known markers before merging of subclusters (**c-e**). (**e**) Dot plot of scaled expression of major cell type marker genes.

Supplementary figure 10

Vascular cell subcluster annotation

a.

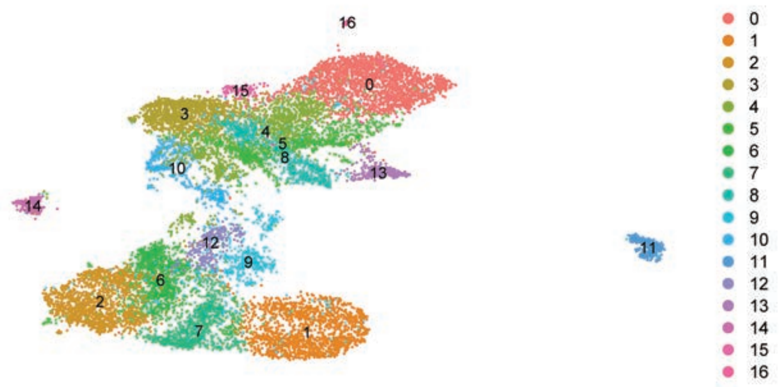

b.

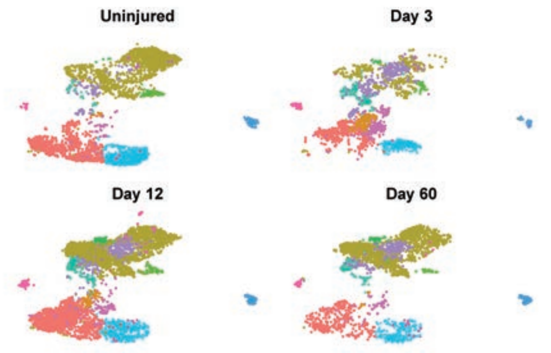

c.

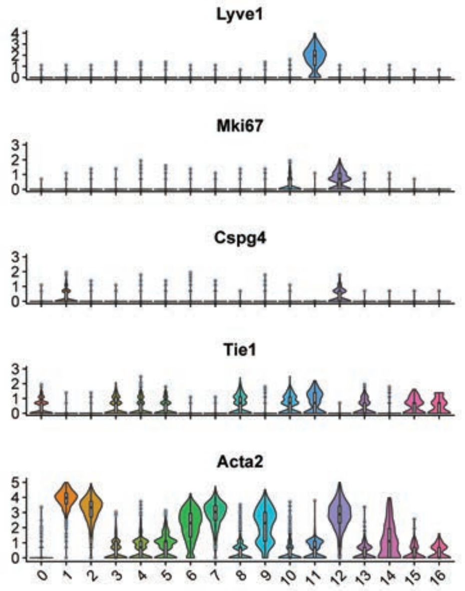

d.

Cluster ID	Cell subtype
0	nEC1
1	PC
2	nSMC1
3	nEC1
4	iEC
5	nEC1
6	nSMC1
7	nSMC1
8	nEC1
9	iSMC
10	dEC
11	LEC
12	dSMCs
13	nEC2
14	Doublets
15	nEC3
16	Doublets

e.

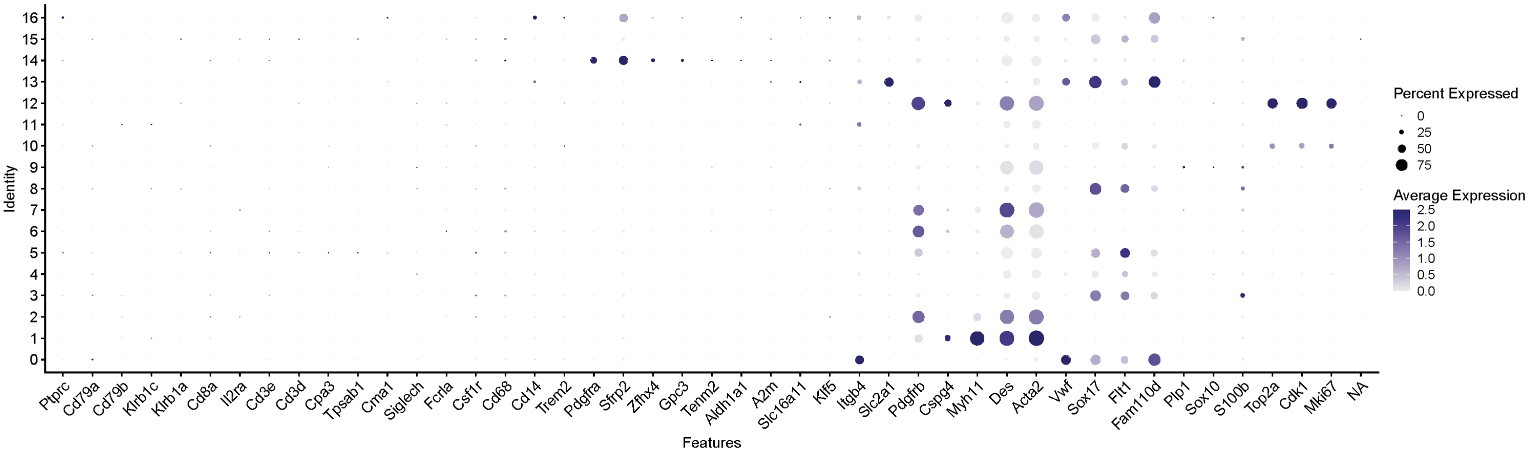

Supplementary Figure 10. Subclustering on vascular cell clusters, at all timepoints (**a upper panel**) and at individual timepoints (A bottom panel). Annotation of vascular cell types based on known markers before merging of subclusters (**b-d**). (**e**) Dot plot of scaled expression of major cell type marker genes.

Supplementary Figure 11

Lymphoid cell subcluster annotation

a.

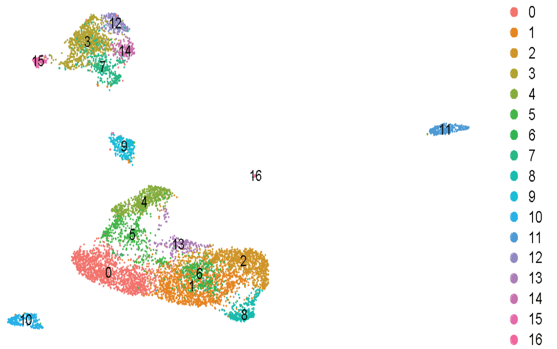

b.

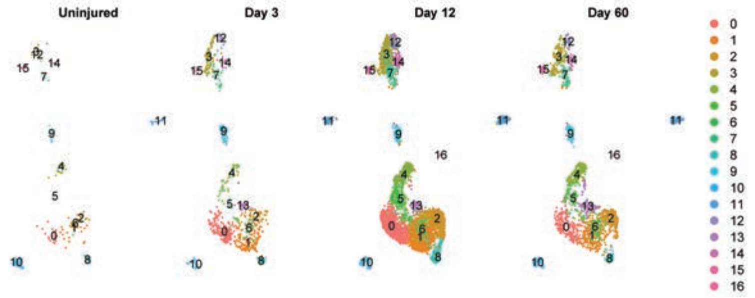

c.

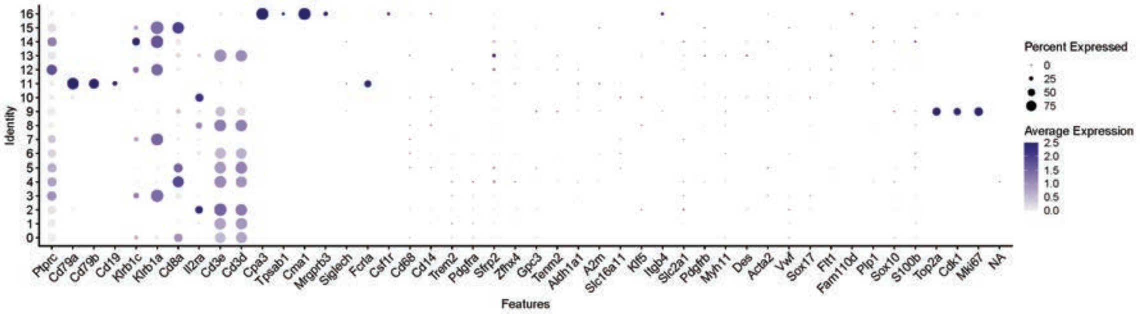

d.

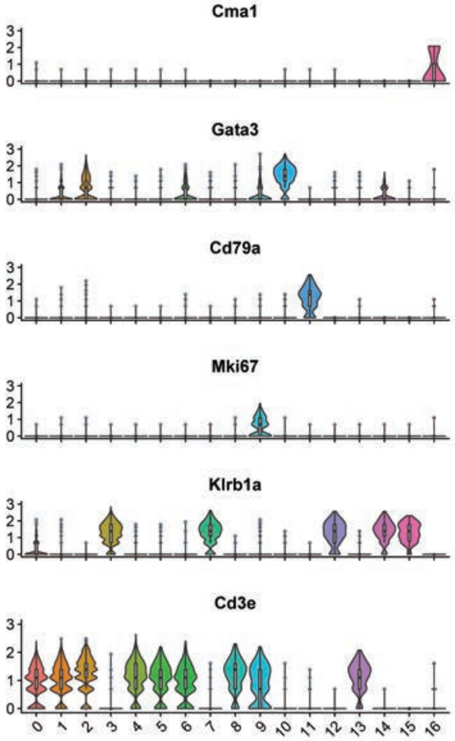

e.

Cluster ID	Cell subtype
0	TCs 1
1	TCs 2
2	TCs 6
3	NK/ILC1 1
4	TCs 4
5	TCs 5
6	TCs 3
7	NK/ILC1 2
8	TCs 7
9	Dividing cells
10	ILC2
11	BCs
12	NK/ILC1 3
13	TCs 8
14	NK/ILC1 4
15	NK/ILC1 5
16	Mast cells

Supplementary Figure 11. Subclustering on lymphoid cell clusters, at all timepoints (**a**) and at individual timepoints (**b**). Annotation of vascular cell types based on known markers before merging of subclusters (**c-d**). Annotation of each cluster ID (**e**).
