# Supplementary material for: scRNA-seq generates a molecular map of emerging cell subtypes after sciatic nerve injury in rats
Source: Commun Biol. 2022 Oct 19;5:1105. doi: 10.1038/s42003-022-03970-0 (PMC9581950; doi:10.1038/s42003-022-03970-0)
Supplement: Supplementary file 4 — Reporting Summary [file 42003_2022_3970_MOESM4_ESM.pdf]

nature portfolio

Corresponding author(s): Ditte Lovatt

Last updated by author(s): Aug 31, 2022

Reporting Summary

Nature Portfolio wishes to improve the reproducibility of the work that we publish. This form provides structure for consistency and transparency in reporting. For further information on Nature Portfolio policies, see our Editorial Policies and the Editorial Policy Checklist.

Statistics

For all statistical analyses, confirm that the following items are present in the figure legend, table legend, main text, or Methods section.

n/a	Confirmed	
☐	☒	The exact sample size (*n*) for each experimental group/condition, given as a discrete number and unit of measurement
☐	☒	A statement on whether measurements were taken from distinct samples or whether the same sample was measured repeatedly
☐	☒	The statistical test(s) used AND whether they are one- or two-sided<br>*Only common tests should be described solely by name; describe more complex techniques in the Methods section.*
☒	☐	A description of all covariates tested
☐	☒	A description of any assumptions or corrections, such as tests of normality and adjustment for multiple comparisons
☐	☒	A full description of the statistical parameters including central tendency (e.g. means) or other basic estimates (e.g. regression coefficient) AND variation (e.g. standard deviation) or associated estimates of uncertainty (e.g. confidence intervals)
☒	☐	For null hypothesis testing, the test statistic (e.g. *F*, *t*, *r*) with confidence intervals, effect sizes, degrees of freedom and *P* value noted<br>*Give P values as exact values whenever suitable.*
☒	☐	For Bayesian analysis, information on the choice of priors and Markov chain Monte Carlo settings
☒	☐	For hierarchical and complex designs, identification of the appropriate level for tests and full reporting of outcomes
☒	☐	Estimates of effect sizes (e.g. Cohen's *d*, Pearson's *r*), indicating how they were calculated

Our web collection on statistics for biologists contains articles on many of the points above.

Software and code

Policy information about availability of computer code

Data collection	No software was used to collect data.
Data analysis	Data normalization and scaling of RNA-seq data: CellRanger (v3.0.0), R (v.3.5) and Seurat (v.3.1).<br>Data mining of RNA-seq data: R (v.3.6) and Seurat (v.3.1)

For manuscripts utilizing custom algorithms or software that are central to the research but not yet described in published literature, software must be made available to editors and reviewers. We strongly encourage code deposition in a community repository (e.g. GitHub). See the Nature Portfolio guidelines for submitting code & software for further information.

Data

Policy information about availability of data

All manuscripts must include a data availability statement. This statement should provide the following information, where applicable:

- Accession codes, unique identifiers, or web links for publicly available datasets
- A description of any restrictions on data availability
- For clinical datasets or third party data, please ensure that the statement adheres to our policy

Data availability. Source data is provided for figures 1f, 1g, 2i, 3h, 3k, 4g, 5c, 5d, 6b, 6j, 7g, 7h, and 7j in Supplementary Data 1. Single-cell RNA-seq data that support the findings of this study is deposited in GEO Omnibus with the accession code (http://www.ncbi.nlm.nih.gov/...).

Human research participants

Policy information about studies involving human research participants and Sex and Gender in Research.

Reporting on sex and gender	N/A
Population characteristics	N/A
Recruitment	N/A
Ethics oversight	N/A

Note that full information on the approval of the study protocol must also be provided in the manuscript.

Field-specific reporting

Please select the one below that is the best fit for your research. If you are not sure, read the appropriate sections before making your selection.

☒ Life sciences ☐ Behavioural & social sciences ☐ Ecological, evolutionary & environmental sciences

For a reference copy of the document with all sections, see nature.com/documents/nr-reporting-summary-flat.pdf

Life sciences study design

All studies must disclose on these points even when the disclosure is negative.

Sample size	We designed the experiment to include both biological replicates and technical replicates. Due to high experimental costs the n was between three and seven animals per experimental group totaling 18 animals in the RNA-seq study (see supplementary table 1).
Data exclusions	Clusters with red blood cells were removed from the processed and mined RNA-seq data (See supplementary Figure 1).
Replication	We analyzed the reproducibility of identifying the same proportions of cell subtypes among different biological samples. This is displayed in a heat-map for each sub-cluster in the manuscript.
Randomization	Rats were randomized into cohorts for surgery.
Blinding	Blinding was not necessary since no subjective scoring was performed.

Reporting for specific materials, systems and methods

We require information from authors about some types of materials, experimental systems and methods used in many studies. Here, indicate whether each material, system or method listed is relevant to your study. If you are not sure if a list item applies to your research, read the appropriate section before selecting a response.

Materials & experimental systems

n/a	Involved in the study	
☒	☐	Antibodies
☒	☐	Eukaryotic cell lines
☒	☐	Palaeontology and archaeology
☐	☒	Animals and other organisms
☒	☐	Clinical data
☒	☐	Dual use research of concern

Methods

n/a	Involved in the study	
☒	☐	ChIP-seq
☒	☐	Flow cytometry
☒	☐	MRI-based neuroimaging

Animals and other research organisms

Policy information about studies involving animals; ARRIVE guidelines recommended for reporting animal research, and Sex and Gender in Research

Laboratory animals	Sprague Dawley rats weighed between 175-250 g at the time of surgery.

Wild animals	Our study did not include wild animals.
Reporting on sex	Sex-based analysis was not the focus of this study.
Field-collected samples	The study did not contain samples collected in the field.
Ethics oversight	For all studies conducted at the Research Labs of Merck & Co., Inc., Kenilworth, NJ, USA, experimental procedures described herein were approved by the Institutional Animal Care and Use Committees, and subjects were housed and tested in AAALAC-accredited facilities.

Note that full information on the approval of the study protocol must also be provided in the manuscript.
